# Distance dependence of enhanced intersystem crossing in BODIPY–nitroxide dyads†

**DOI:** 10.1039/d3sc00589e

**Published:** 2023-05-01

**Authors:** Maximilian Mayländer, Theresia Quintes, Michael Franz, Xavier Allonas, Andreas Vargas Jentzsch, Sabine Richert

**Affiliations:** a Institute of Physical Chemistry, University of Freiburg Albertstraße 21 79104 Freiburg Germany sabine.richert@physchem.uni-freiburg.de; b Laboratoire de Photochimie et d’Ingénierie Macromoléculaires, Institut Jean Baptiste Donnet 3b rue Alfred Werner 68093 Mulhouse Cedex France; c SAMS Research Group, Université de Strasbourg, CNRS, Institut Charles Sadron UPR 22 67000 Strasbourg France vargasjentzsch@unistra.fr

## Abstract

Photogenerated organic triplet–doublet systems have attracted an increasing amount of attention in recent years due to their versatility and suitability for a range of technological applications in the emerging field of molecular spintronics. Such systems are typically generated by enhanced intersystem crossing (EISC) preceded by photoexcitation of an organic chromophore covalently linked to a stable radical. After formation of the chromophore triplet state by EISC, triplet state and stable radical may interact, whereby the nature of the interaction depends on the exchange interaction *J*_TR_ between them. If *J*_TR_ surpasses all other magnetic interactions in the system, molecular quartet states may be formed by spin mixing. For the design of new spintronic materials based on photogenerated triplet–doublet systems, it is crucial to gain further knowledge about the factors influencing the EISC process and the yield of the subsequent quartet state formation. Here we investigate a series of three BODIPY–nitroxide dyads characterised by different separation distances and different relative orientations of the two spin centres. Our combined results from optical spectroscopy, transient electron paramagnetic resonance, and quantum chemical calculations suggest that the chromophore triplet formation by EISC is mediated by dipolar interactions and depends primarily on the distance between the chromophore and radical electrons, while the yield of the subsequent quartet state formation by triplet–doublet spin mixing is influenced by the absolute magnitude of *J*_TR_.

## Abstract 2

A detailed spectroscopic study on a series of photoexcited BODIPY–nitroxides revealed that a high EISC yield does not necessarily imply a high trip-quartet yield as these two processes have a different dependence on dipolar and exchange interactions.

## Graphical abstract

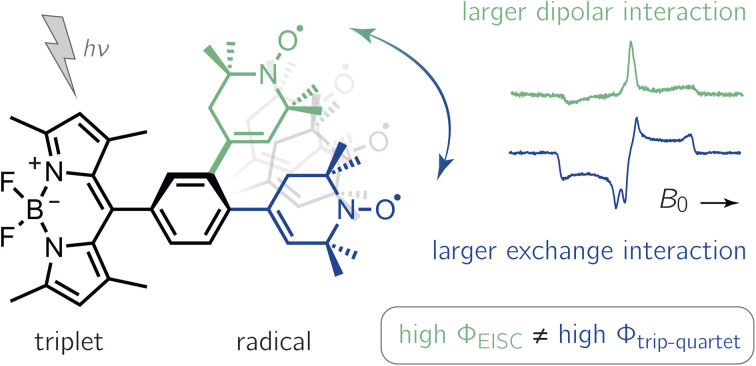

## 1 Introduction

In the past years, the process of enhanced intersystem crossing (EISC)^1–3^ in photoexcited chromophores that are covalently attached to a stable radical has frequently been exploited as a means to access high-spin states of organic chromophores. Examples include the triplet state of perylene diimide (PDI)^4^ or the quartet or quintet states of various chromophore–radical compounds.^5–10^ The latter, apart from being of fundamental interest, may also have promising properties for applications in the emerging field of molecular spintronics. For instance, it has been shown that molecular quartet states of PDI–radical compounds may serve as multi-level spin qubits, *i.e.* qudits, for applications in quantum information science.^11,12^ The increased triplet yield in covalently-linked chromophore–stable radical systems is also attractive for applications as heavy atom free triplet sensitisers for triplet–triplet photon up-conversion or photodynamic therapy.^13–16^

**Fig. 1:**
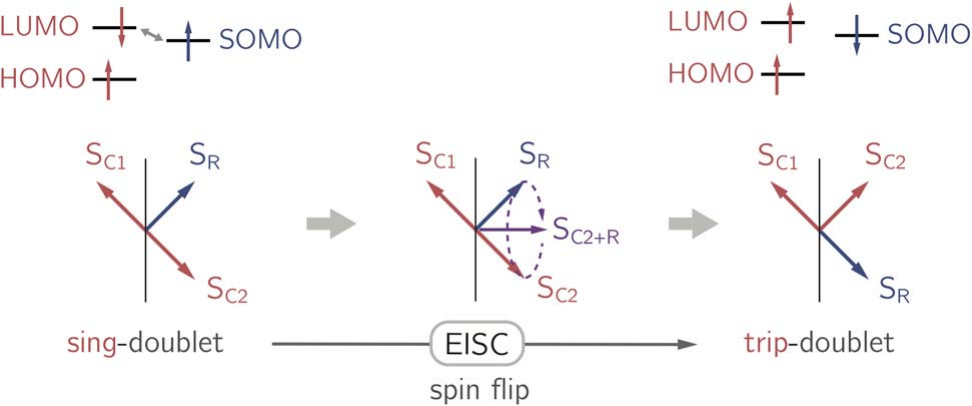
Simplified vector picture of the EISC process generating a local triplet state on the chromophore. The arrows represent the chromophore (C) and radical (R) electrons.

The EISC process is spin-allowed, since the overall doublet spin-multiplicity of the photoexcited chromophore–radical system is conserved. After photoexcitation to the chromophore excited singlet state, the so-called sing-doublet state (D_2_) is converted to a trip-doublet state (D_1_) by spin-exchange.^3,17,18^ The process can be visualised as shown schematically in Fig. 1. The electrons in the LUMO orbital of the chromophore and the SOMO orbital of the radical flip their orientation simultaneously, conserving the overall spin multiplicity while generating a local triplet state on the chromophore. It is known that this process is mediated by electronic interactions, but further mechanistic details have not yet been explored.

For the applicability of photogenerated triplet–doublet systems in future molecular spintronic devices, a high triplet yield will be essential and it is thus of utmost importance to improve our understanding of the factors influencing the yield of the EISC process.

**Fig. 2:**
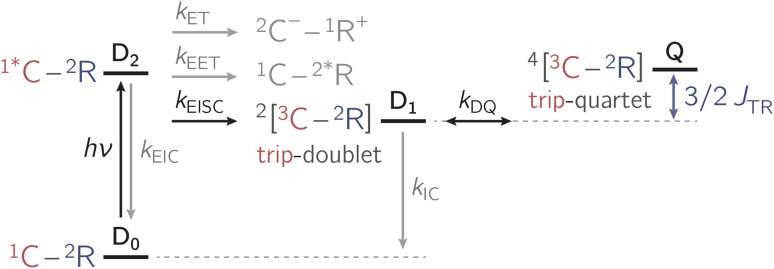
Summary of the photophysical processes occurring in chromophore-radical compounds after photoexcitation. The rate constants *k*_i_ indicate a population transfer between states. Abbreviations: EET – excitation energy transfer; ET – electron transfer; EISC – enhanced intersystem crossing; EIC – enhanced internal conversion; C – chromophore; R – radical; D – doublet; Q – quartet. The numeric superscripts indicate the spin multiplicity.

Apart from EISC, other excited state processes may occur after photoexcitation of the chromophore as shown in Fig. 2. In particular, electron transfer (ET) or excitation energy transfer (EET) may compete with EISC and limit the triplet state formation yield.^4,7,19,20^

Once the chromophore triplet (*i.e.* trip-doublet) state is successfully formed, intersystem crossing to the trip-quartet state may occur. Pure quartet states are only formed if the exchange interaction *J*_TR_ between triplet state and radical surpasses any other magnetic interactions in the system and the ensemble is then said to be within the so-called strong-coupling regime. Different mechanisms have been invoked to explain the spin-forbidden doublet–quartet transition; it is reversible and may for instance be mediated by spin–orbit coupling or dipolar-induced mixing.^2,5,7,8,15,21^ However, the role of the magnitude of *J*_TR_ in this process remained unexplored thus far, largely due to the fact that *J*_TR_ can only be determined experimentally in very rare cases.^22^

Here we investigate a series of three rigid chromophore–radical compounds that differ with respect to the distance and orientation of the chromophore and radical building blocks. The systematic modification of the molecular structure, combined with a detailed spectroscopic and quantum chemical analysis allows us to get further insight into the mechanisms underlying both the EISC process and the subsequent interaction between the triplet and doublet spin centres that governs the formation of photogenerated high-spin states.

**Fig. 3:**
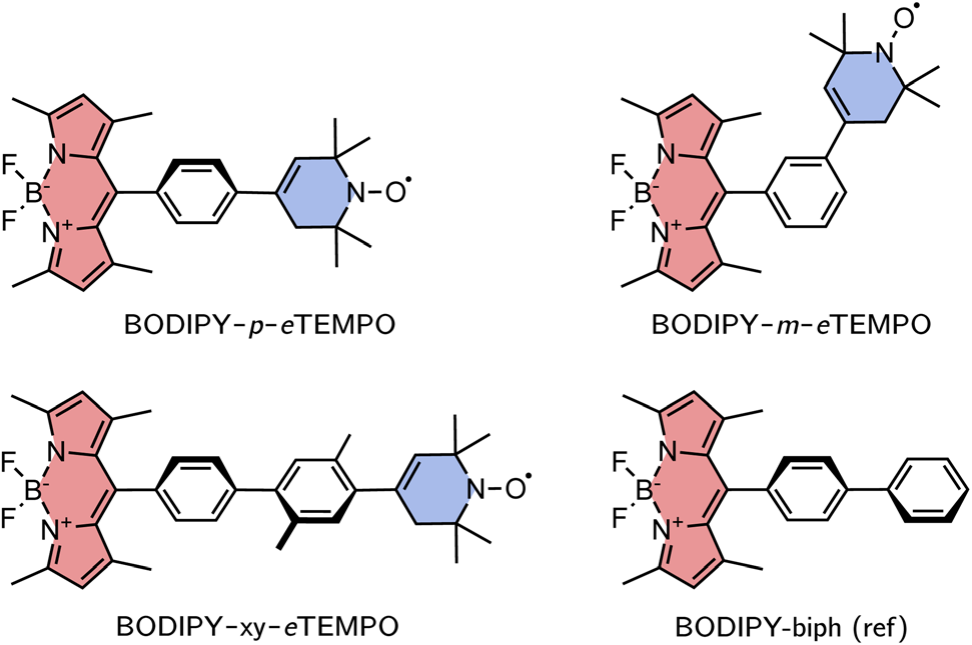
Structures of the investigated BODIPY–*e*TEMPO dyads and the BODIPY reference molecule.

The investigated structures are shown in Fig. 3. 1,3,5,7-Tetramethyl-substituted 4,4-difluoro-4-bora-3*a*,4*a*-diaza-*s*-indacene (BODIPY) was chosen as the chromophore due to its chemical accessibility, high photostability, and its negligible triplet formation from “normal” spin–orbit-coupling induced intersystem crossing.^23–25^ Since the transition dipole moment of the BODIPY chromophore is oriented along the long axis of the core, substitution in *meso* position is thought to limit Förster-type excitation energy transfer as a possible process competing with EISC.

As the radical counterpart, we selected a derivative of the well-known (2,2,6,6-tetramethylpiperidin-1-yl)oxyl (TEMPO) radical with a double bond in the piperidine ring (*cf.*Fig. 3), which we will refer to as *e*TEMPO. Just like TEMPO, this nitroxide radical is particularly stable and synthetically easily accessible.^26,27^ Crucially, and in contrast to a classical TEMPO radical, *e*TEMPO can be connected directly to a phenylene bridge by reliable and well-established Suzuki coupling, enabling a systematic elongation of the linker in regular small steps of 4 Å.^28^ The three BODIPY–*e*TEMPO dyads will be referred to as BODIPY–*p*–*e*TEMPO (or *para*), BODIPY–*m*–*e*TEMPO (or *meta*), and BODIPY–xy–*e*TEMPO (or xy, for xylene). The choice of phenyl and xylyl as spacer units was motivated by our aim to obtain structures that are reasonably rigid and well-defined with only few thermally accessible conformations.

While weak exchange coupling leading to spin polarisation transfer was observed in a previously investigated BODIPY–TEMPO dyad with a flexible linker,^13,29^ transient electron paramagnetic resonance (EPR) spectroscopy combined with quantum chemical calculations on our BODIPY–*e*TEMPO structures reveal that all three triplet–radical compounds are in the strong exchange coupling regime where molecular quartet states are formed. The *para* compound is found to have the highest quartet yield and is characterised by a larger exchange coupling compared to the two other derivatives. In contrast, optical spectroscopy shows that the *meta*-connected dyad, characterised by the shortest separation distance between chromophore and radical, has the largest triplet yield. Our results suggest that quartet state formation in chromophore–radical compounds has to be regarded as two separate processes that depend on different interactions. While a dipolar mechanism seems to govern chromophore triplet formation by EISC, the absolute magnitude of *J*_TR_ appears to play a role in determining the efficiency of trip-quartet population from the trip-doublet state.

## 2 Experimental

### 2.1 Steady-state absorption and fluorescence spectroscopy

Steady-state absorption measurements of the samples in toluene were carried out on a Shimadzu UV-1601 UV-vis spectrometer. For all fluorescence measurements, the samples were diluted substantially, corresponding to absorbances <0.1 at the excitation wavelengths. Steady-state fluorescence spectra were recorded on a FluoroMax-4 fluorimeter from Horiba. The raw spectra were corrected for the spectral sensitivity of the instrument and fluctuations of the excitation light source.

### 2.2 Fluorescence quantum yields and lifetimes

Fluorescence quantum yields were determined using a C11347 absolute photoluminescence quantum yield spectrometer from Hamamatsu Photonics K.K., Japan. The same solutions as prepared for the fluorescence measurements were used. A wavelength of 460 nm was chosen for excitation of the samples and the analysis was performed using the tools provided with the data acquisition software. Fluorescence lifetime measurements were carried out using a FluoTime 100 fluorescence lifetime spectrometer from Picoquant GmbH, Germany. The samples were excited at 470 nm and the scattering light from the excitation source was cut off with the help of a long-pass filter placed in the detection path. The instrument response function was collected (without any filters) using a solution of LUDOX® (colloidal silica) in distilled water. To obtain the fluorescence decay times, iterative re-convolution of the instrument response function with a monoexponential decay function was performed in MATLAB. The model decay function was fit to the experimental data using a least-squares fitting approach (minimisation of the residuals using a built-in trust-region-reflective algorithm).

### 2.3 Singlet oxygen quantum yields

For the singlet oxygen quantum yield measurements, all samples were prepared in toluene solutions with matched absorbances of 0.1 (pathlength 10 mm) at the excitation wavelength of 510 nm. A Continuum Horizon OPO pumped by the third harmonic of a Continuum Surelite laser operated at 10 Hz was used for photoexcitation, whereby the laser intensity was adjusted to amount to 0.5 mJ at the sample using a combination of a half-waveplate and a Glan-Taylor polariser (spot diameter ∼5 mm, pulse length ∼5 ns). The samples (∼2.5 mL) were contained in quartz cuvettes with a pathlength of 10 mm inside a 3D-printed sample holder that can accommodate a cuvette-sized magnetic stirrer. During the measurements, the air saturated solutions were continuously stirred at a speed of 400 RPM to replenish the solution with oxygen.

Singlet oxygen is generated by reaction of molecular triplet states with triplet oxygen dissolved in the solution. The IR light emitted by ^1^O_2_ was collected at 90° using a collimating lens and a back mirror both with a focal length of 50 mm. It passes an interference filter from Spectrogon adapted to the wavelength of 1270 nm before the signal is detected by an IR photomultiplier (Hamamatsu H10330-45) and recorded using a transient digitiser. A total of 256 averages were collected for each sample. The quenching of singlet oxygen is followed by monitoring the transient signal of ^1^O_2_ and the intensity maximum of the signal after laser excitation allows the determination of the singlet oxygen quantum yield by comparison to the behaviour of a known system measured under identical conditions.

The data were referenced against 2,6-diiodo-1,3,5,7-tetramethyl-8-phenyl-BODIPY purchased from BLDpharm (CAS No. 1083009-44-2) with a known singlet oxygen quantum yield of 0.85.^13^

### 2.4 Electron paramagnetic resonance

Continuous wave EPR spectra were recorded at the X-band (9.75 GHz) on a Bruker EMXnano benchtop EPR spectrometer. The modulation frequency was set to 100 kHz and the modulation amplitude to 0.1 mT at a microwave power of 1 mW (20 dB).

For all transient EPR measurements, the samples were prepared with an absorbance of roughly 0.3 at the excitation wavelength of 515 nm, measured in a 2 mm cuvette. For the measurements, the samples in toluene were transferred into quartz EPR tubes with an outer diameter of 3.8 mm (inner diameter of 3 mm). The solutions were rapidly frozen in liquid nitrogen before insertion into the EPR resonator for the measurement.

All transient EPR measurements were performed at X-band frequencies (9.75 GHz) on a Bruker ELEXSYS E580 spectrometer equipped with a Bruker ER4118X-MD-5 resonator. During the measurement, the sample was kept at a constant temperature of 80 K using an Oxford Instruments nitrogen gas-flow cryostat (CF 935). The samples were excited through the front window of the cryostat and resonator with depolarised light at 515 nm. The excitation energy was ∼1 mJ at a repetition rate of 50 Hz (pulse duration ∼5 ns).

The transient EPR spectra were acquired in direct detection mode using the transient recorder and a microwave power of 1.5 mW (20 dB). Any positive signals thus corresponds to an absorptive transition and any negative signals to an emissive one. Typically, for every magnetic field value, a time trace with 4096 points was recorded using a time base of 4 ns. After data acquisition, the 2D spectra were baseline-corrected in both dimensions using a lab-written MATLAB routine. The shape of the transient EPR spectra was found not to change significantly over the course of the excited state lifetime. The spectra shown in the figures have been averaged over a time window from 0.2 μs to 1 μs after laser excitation. All EPR spectra were frequency-corrected to 9.75 GHz and field-corrected using a carbon fiber standard with *g* = 2.002644.^30^

### 2.5 Electrochemistry

To determine the half wave potentials of BODIPY and *e*TEMPO, cyclic voltammograms were recorded using an Autolab PGstat101 potentiostat from Metrohm. A platinum wire with a diameter of 0.25 mm was used as the working electrode, whereas a platinum gauze (80 mesh 6 × 7 mm) served as the counter electrode. A silver/silver chloride wire was used as pseudo reference electrode and the measurements were performed in a quartz glass cell (1 mm path length) bought from ALS Japan. The analyte solutions in *o*-dichlorobenzene had a concentration of roughly 1 mM and were purged with argon for at least 10 minutes prior to the measurement. The reported redox values were internally referenced against ferrocene measured before and after each experiment.

## 3 Results

### 3.1 Synthesis

The synthetic protocols for the three BODIPY–*e*TEMPO dyads as well as the *e*TEMPO and BODIPY reference compounds are described in the Electronic Supporting Information (ESI).† The identity and purity of all compounds and synthetic intermediates was characterised using standard techniques as also presented in the ESI.†

### 3.2 UV-vis spectroscopy

**Fig. 4:**
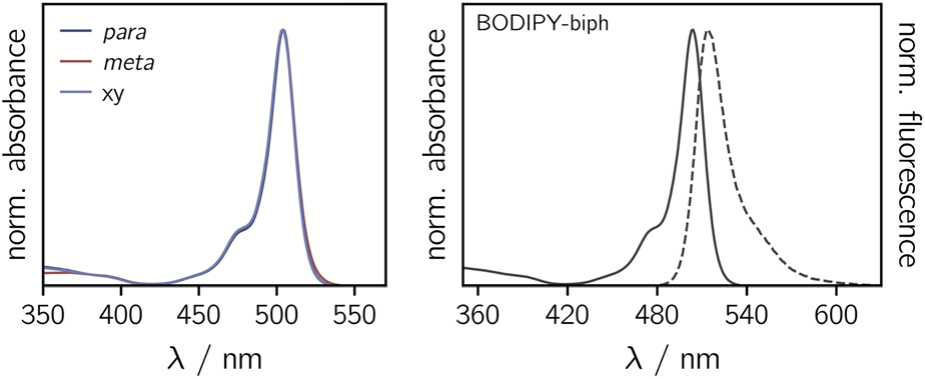
Normalised UV-vis absorption spectra of the three BODIPY–*e*TEMPO dyads (left) and absorption and fluorescence spectra of the BODIPY-biph reference compound (right).

The UV-vis absorption spectra of the BODIPY–*e*TEMPO dyads are shown in Fig. 4. All spectra are virtually identical to the known spectrum of 1,3,5,7-tetramethyl-BODIPY.^31^ This is due to the fact that the *e*TEMPO radical absorbs only very weakly in the visible range with a molar absorption coefficient of *ε* = 21.2 M^−1^ cm^−1^ at its absorption maximum of 458 nm. The corresponding data are shown in Fig. S2.† It can also be seen that, compared to classical TEMPO, the absorption maximum of *e*TEMPO is slightly blue-shifted by ∼10 nm and the absorption band is characterised by some additional vibrational structure due to the increased structural rigidity. Similar to TEMPO, the absorption tail on the red edge of the spectrum reaches out to about 640 nm.

Table 1 summarises the fluorescence properties of the investigated compounds. Compared to the reference BODIPY compound, the fluorescence quantum yields of the *para* and *meta e*TEMPO-compounds are reduced from 50% to 25% and 15%, respectively. However, when a further xylene spacer is added in BODIPY–xy–*e*TEMPO, the original fluorescence of 50% is found to recover. The same trend is reflected in the fluorescence lifetimes. The excited state decay times measured by time-correlated single photon counting amount to 2.83 ns for BODIPY-biph and 2.89 ns for BODIPY–xy–*e*TEMPO, while for BODIPY–*p*–*e*TEMPO and BODIPY–*m*–*e*TEMPO we obtained S_1_ state decay time constants of 1.54 ns and 0.88 ns, respectively. The experimental data can be found in Fig. S3† in the ESI.

**Table 1:** Overview of the photophysical properties in toluene at 295 K. *Φ*_F_: fluorescence quantum yield; *τ*_S_1__: fluorescence lifetime; *Φ*_Δ_: singlet oxygen quantum yield

Compound	*Φ*_F_	*τ*_S_1__/ns	*Φ*_Δ_
BODIPY–*p*–*e*TEMPO	0.25	1.54	0.12
BODIPY–*m*–*e*TEMPO	0.15	0.88	0.23
BODIPY–xy–*e*TEMPO	0.50	2.89	0.02
BODIPY-biph (ref)	0.50	2.83	0.02

To estimate the triplet formation (*i.e.* EISC) yields of the compounds, measurements of the singlet oxygen quantum yields were carried out. The obtained values are also listed in Table 1 and the raw data are shown in the ESI (Fig. S5†). As one might have anticipated, it can be seen that a reduced fluorescence quantum yield translates to an increased triplet formation yield in all cases. BODIPY–*m*–*e*TEMPO, which had the lowest *Φ*_F_, has the highest *Φ*_EISC_ of 23%. Again it is observed that the BODIPY-biph reference compound and BODIPY–xy–*e*TEMPO behave almost the same. Both are found to have a triplet yield of only a few percent (2–3% within error), while BODIPY–*p*–*e*TEMPO is characterised by a triplet yield of 12%.

### 3.3 EPR spectroscopy

To determine their magnetic properties, the three BODIPY–*e*TEMPO dyads were characterised by EPR spectroscopy with and without photoexcitation. Fig. S7† in the ESI shows the continuous wave (cw) EPR spectra recorded in the dark at room temperature. The spectra all show the typical three-line pattern expected for nitroxide radicals. Only small differences in the intensity of the third nitroxide line can be discerned, which reflect differences in the rotational correlation times due to variations in the molecular size. For an accurate determination of the nitroxide **g** tensor, we performed a global fit of the room temperature cw EPR spectrum and a frozen solution (80 K) Q-band (34 GHz) pulse EPR spectrum of *e*TEMPO. From the fits to the data shown in the ESI (see Fig. S8†) principal **g** tensor values of [2.0103 2.0070 2.0025] were obtained.

**Fig. 5:**
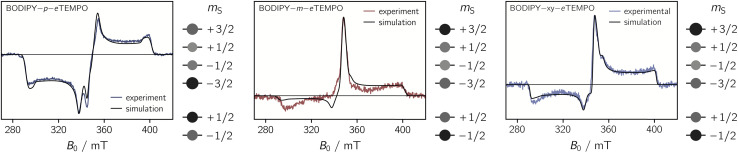
Transient cw EPR spectra of the three BODIPY–*e*TEMPO dyads together with numerical simulations of the data. The relative populations of the doublet and quartet sublevels, obtained from the simulations, are indicated by circles of different sizes.

The transient continuous wave EPR (trEPR) spectra of the three dyads are shown in Fig. 5. Compared to the triplet EPR spectrum of a commercial diiodo-BODIPY derivative (*i.e.* 2,6-diiodo-1,3,5,7-tetramethyl-8-phenyl-BODIPY), the spectra of all three dyads are found to be significantly narrower. While the triplet spectrum spans about 2200 G (see Fig. S10†), the spectra of the dyads have a width of only ∼1150 G. The order of absorptive (*a*) and emissive (*e*) transitions in the triplet state EPR spectrum, *i.e.* its *eeeaaa* multiplet polarisation, is conserved in the spectra of the dyads. In addition, the spectra are characterised by a prominent line in the center of the spectrum. This central transition is characteristic for photogenerated quartet states and corresponds to the quartet 
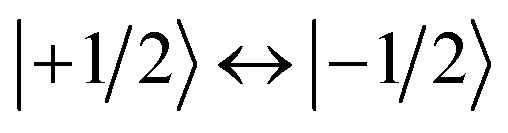
 transition.

Simulations reveal that the BODIPY triplet state EPR spectrum can be reproduced well assuming zero-field splitting parameters of *D*_T_ = 2980 MHz, *E*_T_ = −660 MHz, and an isotropic *g* value of 2.0068 (see Fig. S9†). These triplet state parameters, as well as the **g** tensor of the *e*TEMPO building block, were then kept fixed for the simulation of the trEPR spectra of the BODIPY–*e*TEMPO dyads. Although this simulation strategy leaves very little degrees of freedom, we were able to obtain good fits to the experimental data which confirms the quartet nature of the dyad spectra.

The trEPR spectra of all three dyads can be simulated with almost the same set of parameters and only minor differences in the quartet state populations as visualised in Fig. 5. These small differences cause the inversion of the central quartet line from emissive for BODIPY–*p*–*e*TEMPO to absorptive for the two other dyads. All compounds are clearly in the strong coupling regime where the value of *J*_TR_ has no influence any more on the spectral shape. The latter can thus not be determined experimentally. An overview of all simulation parameters is provided in the ESI.†

### 3.4 Quantum chemical calculations

Previously, we have shown that *ab initio* calculations in combination with effective Hamiltonian theory allow us to predict qualitatively correct exchange coupling parameters in the electronically excited state of chromophore–radical compounds as well as reliable parameter trends.^32^ Building on this methodology, we calculated *J*_TR_ for the three investigated BODIPY–*e*TEMPO dyads as well as the individual contributions *J*_*ij*_ contributing to *J*_TR_. Computational details are outlined in the ESI† and a visualisation of the localised CAS (3,3) orbitals of all three investigated compounds is provided in Fig. S13.†

From our previous work it is known that *J*_TR_ in chromophore–radical systems can, to a first approximation, be expressed as1
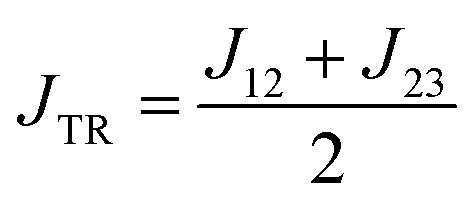
where the indices 1, 2, and 3, refer to the electrons in the HOMO, SOMO, and LUMO orbitals, respectively. The results from these calculations, using the three-electron-three-center Heisenberg–Dirac–Van-Vleck Hamiltonian of the form2
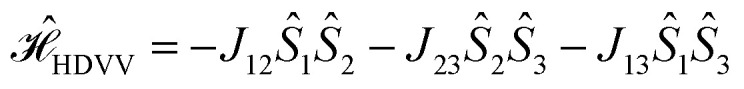
are summarised in Table 2.

**Table 2:** Calculated exchange coupling constants and individual contributions to *J*_TR_

	*J*_12_	*J*_13_	*J*_23_	*J*_TR_/cm^−1^
BODIPY–*p*–*e*TEMPO	−0.0857	+11 207	+0.0436	−0.0211
BODIPY–*m*–*e*TEMPO	−0.0019	+11 111	−0.0226	−0.0123
BODIPY–xy–*e*TEMPO	+0.0085	+11 173	−0.0334	−0.0124

It can be seen from the data that all BODIPY–*e*TEMPO dyads are antiferromagnetically coupled, meaning that the trip-doublet state is lower in energy than the trip-quartet state. As a consequence, the spin-allowed transition from the trip-doublet D_1_ state to the D_0_ ground state might be particularly fast in these molecules and might have a negative impact on the efficiency of trip-quartet state formation. This could be responsible for the low quartet yield observed for all three BODIPY–*e*TEMPO dyads, but does not explain any differences between them.

By looking at the relative magnitudes of the computed *J*_TR_ values, we can further note that the largest absolute coupling is calculated for BODIPY–*p*–*e*TEMPO. The absolute magnitude is about a factor of two larger than that predicted for both BODIPY–*m*–*e*TEMPO and BODIPY–xy–*e*TEMPO. Interestingly, it is observed that the *meta* and xylene-linked derivatives are characterised by near-identical *J*_TR_ values although the distance between the chromophore and radical electrons is very different in these two structures (0.95 nm *vs.* 1.5 nm). Regarding the individual contributions to *J*_TR_, it can be seen that all absolute magnitudes are smaller for the *meta* compound and that both *J*_12_ and *J*_23_ have a negative sign, while in the *para* and xylene-linked structures one of the two contributions is positive.

In some previously investigated chromophore–radical compounds, a change in the sign of *J*_TR_ was observed when comparing structures with the radical connected in *para* and *meta* positions.^7,33,34^ Our calculations reproduce the sign change in these systems correctly,^32,35^ demonstrating the reliability of the chosen approach to calculate *J*_TR_. Comparing the relevant individual exchange interactions, *J*_12_ and *J*_23_, computed for the investigated BODIPY–nitroxide dyads, it can be seen that a change in sign is also observed in our case for one of the two contributions. However, the relative magnitude of this contribution is not significant enough for *J*_TR_ to become positive. Investigations into the factors controlling the sign and magnitude of the individual contributions to *J*_TR_ are currently underway in our group and promise to reveal the physical origin of this observation.

## 4 Discussion

### 4.1 Competing excited state processes

In order to establish whether other excited state processes, apart from internal conversion, are competing with EISC, the time constants for Förster-type excitation energy transfer (EET) and the driving forces for photoinduced electron transfer (ET) were calculated as detailed in the ESI.†

Regarding the likelihood of excitation energy transfer, our calculations reveal that EET could be feasible in such kind of molecules although the *e*TEMPO radical absorbs only weakly in the visible range. The fluorescence quantum yield of the BODIPY chromophore of 50% is relatively high and the spectral overlap of the BODIPY emission and the *e*TEMPO absorption spectra is significant. However, the Förster rate constants further depend on (i) the mutual orientation of the transition dipole moments quantified by the parameter *κ*^2^, which can take any values from 0 (perpendicular) to 4 (collinear), as well as (ii) the distance between chromophore and radical.

TD-DFT calculations, as shown in the ESI (see Fig. S11†), demonstrate that the transition dipole moment is oriented along the long axis of the BODIPY chromophore and therefore perpendicular to the chromophore–radical bonding axis. In addition, the transition of *e*TEMPO in the visible range also has its transition dipole moment oriented perpendicular to the bonding axis. When calculating the *κ*^2^ values based on the computed minimum structures of the dyads, values close to zero were obtained for BODIPY–*p*–*e*TEMPO and BODIPY–xy–*e*TEMPO, indicating a near perpendicular orientation of the transition dipole moments. Due to the twisted molecular structure, a higher *κ*^2^ value of 0.33 was obtained for BODIPY–*m*–*e*TEMPO. Considering also the chromophore-radical separation distances of 1.1 nm, 0.95 nm and 1.5 nm, this translates to calculated EET time constants of 27 ns, 3 ns and 159 ns for the *para*, *meta* and xylene-connected dyads, respectively. Since these time constants are significantly greater than the excited state lifetime of the parent BODIPY chromophore, excitation energy transfer can safely be neglected in these dyads. The results are summarised in Table 3.^36^

**Table 3:** Transition dipole moment orientation *κ*^2^, donor–acceptor distance *r*_DA_ and calculated time constants for Förster-type excitation energy transfer *τ*_EET_

Compound	*κ* ^2^	*r*_DA_/nm	*τ*_EET_/ns
BODIPY–*p*–*e*TEMPO	0.08	1.1	27
BODIPY–*m*–*e*TEMPO	0.33	0.95	3.0
BODIPY–xy–*e*TEMPO	0.10	1.5	159

To estimate the feasibility of electron transfer from the *e*TEMPO radical to BODIPY, the driving forces for charge separation −Δ*G*_CS_ and reorganisation energies *λ* were calculated. The redox potentials entering the calculation were measured by cyclic voltammetry as shown in Fig. S4† in the ESI, while the energy of the first excited singlet state of BODIPY, *E*_00_, was determined from the crossing point of the absorption and fluorescence spectra and amounts to 2.44 eV (see also Fig. 4). The distances and van-der-Waals radii required for the calculation of the Coulomb and solvent correction terms were obtained from quantum chemical models of the structures. As a result from these calculations it is found that −Δ*G*_CS_ is slightly negative for all three dyads while *λ* amounts to 0.27 ± 0.02 eV, meaning that charge separation is not expected to occur spontaneously and can likely be excluded for the compounds investigated here.

In summary, we can thus assume that, apart from EISC and fluorescence emission, internal conversion is the only excited singlet state deactivation pathway active in the investigated BODIPY–*e*TEMPO dyads.

### 4.2 EISC and quartet state formation

By combining our knowledge on the EISC yields of the different BODIPY–*e*TEMPO compounds with insights from quantum chemical calculations, we can rationalise which factors influence the efficiency of triplet formation and subsequent trip-quartet state formation in these molecules.

Different mechanisms for EISC have been proposed in the literature; however, all of them comply with the fact that EISC is mediated by electronic interactions between the chromophore and radical electrons.^3,17,18,37^ This implies essentially that the transition to the chromophore triplet state could in principle be mediated either by dipolar or exchange interactions.

The highest EISC yield was observed for BODIPY–*m*–*e*TEMPO. However, since BODIPY–*m*–*e*TEMPO was found to have a significantly lower *J*_TR_ than BODIPY–*p*–*e*TEMPO, the higher triplet yield in BODIPY–*m*–*e*TEMPO cannot be due to a larger (absolute magnitude of) *J*_TR_. The absence of any clear dependence of the EISC yield on the exchange coupling suggests a mechanism of dipolar nature.

If the mechanism is of dipolar nature, its efficiency should depend purely on the distance between the chromophore and radical electrons. Assuming point dipoles, the separation distances can be translated into dipolar coupling strengths according to3
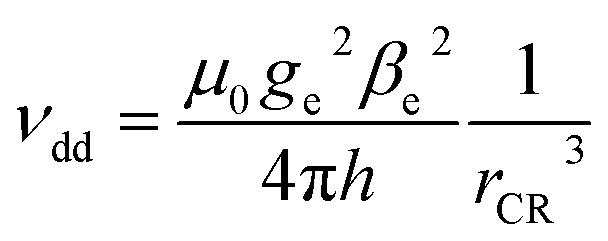
where *μ*_0_ is the vacuum permeability, *g*_e_ the free electron *g* value, *β*_e_ the Bohr magneton, and *h* the Planck constant. For the distances of 1.1 nm, 0.95 nm and 1.5 nm in the *para*, *meta*, and xylene-linked structures, we obtain dipolar coupling strengths of 39 MHz, 60 MHz and 15 MHz. Since these coupling strengths correlate well with the observed EISC yields of 12%, 23%, and 2%, we propose that the EISC process is mediated by dipolar interactions.

The second step after triplet formation by EISC is the doublet-quartet mixing leading to trip-quartet state formation. Different interactions have been proposed already in the literature that mediate this reversible transition, such as spin–orbit coupling or dipolar-induced mixing,^2,5,7,8,15,21^ but the role of the magnitude of *J*_TR_ in this process has not been discussed in detail so far since *J*_TR_ can frequently not be determined experimentally. However, as we have shown previously,^32^ our quantum chemical approach allows reliable predictions of the relative magnitudes and signs of *J*_TR_ which will eventually help us to get further insight.

The EISC process limits the triplet formation yield. However, a high triplet yield does not necessarily translate into a high trip-quartet yield. Although the *meta* compound is found to have the highest triplet yield, our transient EPR experiments suggest that the quartet yield is significantly higher in the *para* compound: considering a comparable measurement time and identical instrumental settings, the signal-to-noise ratio of the quartet state spectrum of the BODIPY–*p*–*e*TEMPO compound is about a factor of two higher as compared to the BODIPY–*m*–*e*TEMPO compound.

A low transient EPR signal could also be due to efficient relaxation from the trip-doublet state D_1_ to the ground state D_0_. However, since all three BODIPY–*e*TEMPO compounds are antiferromagnetically coupled, fast relaxation cannot explain the observed differences in the EPR signal intensities which suggest a higher quartet state formation yield for the *para* compound than for the *meta* compound. Energetic differences can also not be invoked as an explanation, since, to a first approximation, the energies of the D_2_ and D_1_ states (see Fig. 2) only depend on the energetic states of the chromophore and are therefore near-identical for all investigated dyads. As evinced by the simulations of the trEPR spectra, also any other magnetic interactions, such as the zero-field splitting interaction, only depend on the chromophore and are thus identical for all three dyads.

Consequently, it seems that the only difference between the three BODIPY–*e*TEMPO compounds is the exchange coupling strength between the electrons. We therefore suggest that the larger quartet state yield in the BODIPY–*p*–*e*TEMPO compound could be explained by the larger absolute value of *J*_TR_ as compared to BODIPY–*m*–*e*TEMPO and BODIPY–xy–*e*TEMPO. This effect can be understood when considering that the magnitude of *J*_TR_ influences the efficiencies and relative importance of the different mechanisms responsible for doublet-quartet spin mixing. Dipolar-induced mixing^5,7,21^ becomes less efficient with increasing |*J*_TR_| as the rate has an inverse dependence on the energy gap between the trip-doublet and trip-quartet states. On the other hand, SOC-induced mixing^2,5,21^ has no clear dependence on |*J*_TR_| and might therefore become dominant in cases where dipolar-induced mixing is inefficient.

We thus suggest that the different magnitudes of |*J*_TR_| in the three dyads result in differences in the efficiencies of the possible doublet–quartet mixing mechanisms. Mechanistic differences are also suggested by the observation of different polarisation patterns in the trEPR spectra of the three compounds. As it was shown in Fig. 5, the spectrum of the BODIPY–*p*–*e*TEMPO compound clearly differs from that of the other two compounds, which are characterised by near-identical polarisation patterns and a near-identical *J*_TR_.

## 5 Conclusions

Three BODIPY–*e*TEMPO dyads, that differ with respect to the distance and orientation of the chromophore and radical moieties, were investigated by optical spectroscopy, transient EPR spectroscopy as well as quantum chemical methods. The structural differences were found to result in different EISC and quartet state formation yields: While the *meta*-linked compound was found to have a higher triplet state formation yield, its quartet state signal, measured by trEPR, is considerably weaker than that of the *para*-connected derivative. In the *meta* structure, the radical and chromophore electrons are significantly closer, but the computed exchange coupling value is smaller by roughly a factor of two compared to the *para* compound.

Considering all three dyads, we observed a clear correlation between the EISC yield and the dipolar coupling strength calculated from the distance between chromophore and radical electrons. The combined analysis of all results suggests that the dipolar interaction between chromophore and radical electrons mediates the transition from the sing-doublet (D_2_) to the trip-doublet (D_1_) state in the investigated BODIPY–*e*TEMPO dyads. The magnitude of the exchange coupling interaction, on the other hand, seems to play a role in determining the mechanism responsible for the subsequent transition from the trip-doublet to the trip-quartet state.

This study demonstrates that a high EISC yield does not necessarily translate to a high trip-quartet population in photogenerated triplet–doublet systems. Quartet state formation has to be regarded as a two-step process, whereby the two consecutive steps depend on different interactions that are not necessarily related.

## Data availability

The data supporting the findings of this study are available within the article and in the ESI.†

## Author contributions

Spectroscopic characterisation of the compounds, EPR data acquisition and analysis, draft editing and data visualisation M. M.; EPR data analysis and simulations, draft editing and data visualisation T. Q.; quantum chemical calculations and analysis including determination of the excited state exchange couplings, draft editing and data visualisation M. F.; construction of the setup for the determination of singlet oxygen quantum yields X. A.; synthesis and characterisation of the compounds, electrochemical measurements A. V. J.; conceptualisation, project administration, funding acquisition, supervision of the optical and EPR experiments, original draft writing S. R.

## Conflicts of interest

There are no conflicts to declare.

## Supplementary Material

SC-014-D3SC00589E-s001
