# Supplementary material for: Distance dependence of enhanced intersystem crossing in BODIPY–nitroxide dyads
Source: Chem Sci. 2023 May 1;14(20):5361–8. doi: 10.1039/d3sc00589e (PMC10207891; doi:10.1039/d3sc00589e)
Supplement: SC-014-D3SC00589E-s001 [file SC-014-D3SC00589E-s001.pdf]

Electronic Supplementary Material (ESI) for Chemical Science.
This journal is © The Royal Society of Chemistry 2023

Supporting Information for

Distance dependence of enhanced intersystem crossing in BODIPY–nitroxide dyads

Maximilian Mayländer,[1] Theresia Quintes,[1] Michael Franz,[1] Xavier Allonas,[2] Andreas Vargas Jentzsch,[3]* Sabine Richert [1]*

[1] *Institute of Physical Chemistry, University of Freiburg, Albertstraße 21, 79104 Freiburg, Germany*
[2] *Laboratoire de Photochimie et d'Ingénierie Macromoléculaires, Institut Jean Baptiste Donnet, 3b rue Alfred Werner, 68093 Mulhouse Cedex, France*
[3] *SAMS Research Group, Université de Strasbourg, CNRS, Institut Charles Sadron UPR 22, 67000 Strasbourg, France*

* E-mail: vargasjentzsch@unistra.fr, sabine.richert@physchem.uni-freiburg.de

Table of Contents

1 Synthetic procedures **S1**
1.1 General methods S2
1.2 Synthetic protocols S2

2 Spectroscopic characterisation **S8**
2.1 Determination of the molar absorption coefficient of *e*TEMPO S8
2.2 Determination of the fluorescence lifetimes S8
2.3 Experimental determination of the redox potentials S9
2.4 Determination of the singlet oxygen quantum yields S9
2.5 Calculation of the Förster energy transfer rate constant S10
2.6 Calculation of the driving forces for electron transfer S11
2.7 Dark state EPR spectra S13
2.8 Simulations of the transient EPR spectra S13

3 Quantum chemical calculations **S16**
3.1 Structures and transition dipole moments S16
3.2 HOMO and LUMO orbitals of BODIPY S16
3.3 Calculation of the inner sphere reorganisation energy S17
3.4 Exchange coupling calculations S17

4 NMR spectra **S19**

5 HRMS data **S31**

List of Figures

S1 Overview of the synthetic procedure S1
S2 Molar absorption coefficient of *e*TEMPO S8
S3 Fluorescence lifetime measurements S8
S4 Determination of the redox potentials S9
S5 Determination of the singlet oxygen quantum yield S10
S6 Spectral overlap of chromophore emission and radical absorption S10
S7 Continuous wave EPR spectra S13
S8 Determination of the nitroxide **g** tensor S14
S9 Transient cw EPR spectrum of the BODIPY triplet state S14
S10 Transient cw EPR spectra of the BODIPY−*e*TEMPO dyads S15
S11 TDM orientations and distances S16
S12 HOMO/LUMO orbitals of BODIPY S16
S13 Localised CAS(3,3) orbitals S18
S14 ^{1}H NMR spectrum of *para*-I-BODIPY and BODIPY-*p*-*e*TEMPO S19
S15 ^{1}H and APT (^{13}C) NMR spectra of *meta*-I-BODIPY S20
S16 ^{11}B and ^{19}F NMR spectra of *meta*-I-BODIPY S21
S17 ^{1}H and APT (^{13}C) NMR spectra of BODIPY-*m*-*e*TEMPO S22
S18 ^{11}B and ^{19}F NMR spectra of BODIPY-*m*-*e*TEMPO S23
S19 ^{1}H and APT (^{13}C) NMR spectra of compound S9 . . . . S24
S20 ^{11}B and ^{19}F NMR spectra of compound S9 . . . . S25
S21 ^{1}H NMR spectra of compounds S8 and BODIPY-xy-*e*TEMPO S26
S22 ^{11}B and ^{19}F NMR spectra of BODIPY-xy-*e*TEMPO S27
S23 ^{1}H NMR spectra of ph-*e*BODIPY and BODIPY-biph S28
S24 ^{13}C and DEPT-135 NMR spectra of BODIPY-biph S29
S25 ^{11}B and ^{19}F NMR spectra of BODIPY-biph S30
S26 HRMS-ESI analysis for BODIPY-*p*-*e*TEMPO and BODIPY-*m*-*e*TEMPO S31
S27 HRMS-ESI analysis for BODIPY-*xy*-*e*TEMPO S32

1 Synthetic procedures

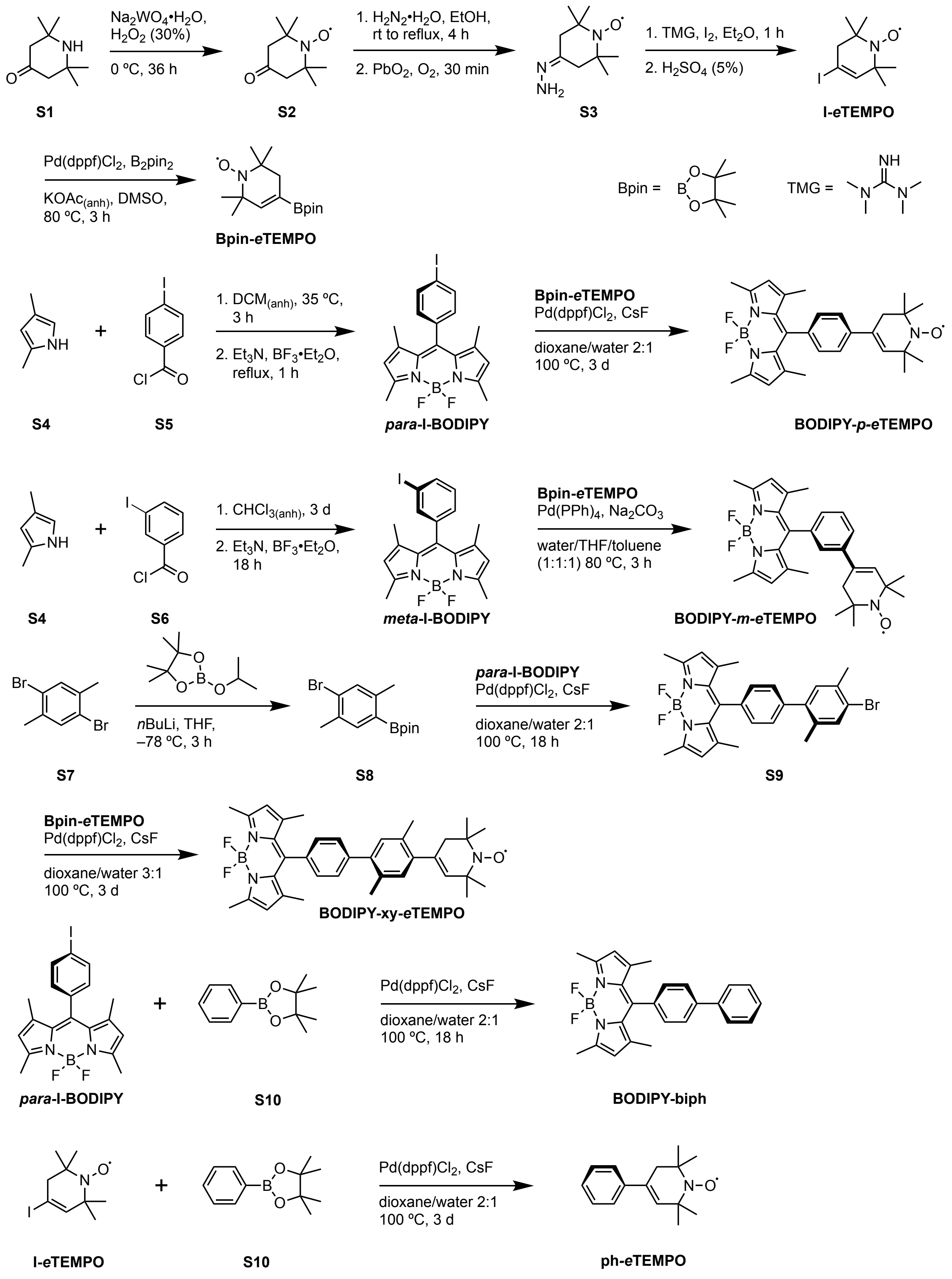

Figure S1: Overview of the procedure employed for the synthesis of the three BODIPY−*e*TEMPO dyads.

1.1 General methods

All reactions were performed under an argon atmosphere unless otherwise indicated. All reagents and solvents were purchased at the highest commercial quality and used without further purification unless otherwise noted. Dry solvents were obtained using a double column SolvTech purification system. Thin layer chromatography was performed with TLC silica on aluminium foil (Silica gel/UV254, Aldrich). In most cases, irradiation using a Bioblock VL-4C UV-lamp (6 W, 254 nm and/or 365 nm) was used as well as suitable TLC stains for visualisation. Preparative adsorption flash column chromatography was performed using silica gel (60 Å, 230–400 mesh, 40–63 μm, Sigma-Aldrich). ^{1}H NMR and ^{13}C NMR spectra were recorded on a Bruker Avance III HD 400 MHz spectrometer equipped with a BBFO probe at 298 K. The spectra were internally referenced to the residual proton solvent signal. For ^{1}H NMR and ^{13}C assignments, the chemical shifts are given in ppm. Coupling constants J are listed in Hz. Ultra performance liquid chromatography coupled to mass spectrometry (UPLC-MS) was carried out on a Waters Acquity UPLC-SQD apparatus equipped with a PDA detector (190−500 nm, 80 Hz), using a reverse phase column (Waters, BEH C18 1.7 μm, 2.1 mm × 50 mm), and the MassLynx 4.1 − XP software with a gradient (water-acetonitrile + 0.1% formic acid) as eluent. High-resolution mass spectra (HRMS) were recorded on a Thermo Fisher Scientific Exactive mass spectrometer with an orbitrap analyser using either atmospheric pressure chemical ionisation (APCI) or electrospray ionisation (ESI).

1.2 Synthetic protocols

Compound S2 (4-oxo-TEMPO)

This compound was prepared following a reported procedure.[1] Briefly, at 0 °C hydrogen peroxide (30% v/v, 3.29 eq., 60 g, 60 mL, 529.19 mmol) was added dropwise (over 10 minutes) to a solution of 2,2,6,6-tetramethylpiperidin-4-one (S1, 1 eq., 25 g, 161.04 mmol) and sodium tungstate dihydrate ($Na_2WO_4 \cdot H_2O$, 0.04 eq., 2.13 g, 6.44 mmol) in water (125 mL). The reaction mixture was left on ice and allowed to slowly warm up to room temperature under continuous stirring 36 h. After addition of brine (50 mL) the aqueous layer was extracted with methyl *tert*-butyl ether (MTBE, 3×150 mL). The combined organic layers were dried over anhydrous Na_2SO_4 and the solvent was removed in vacuo. The crude compound was purified by column chromatography (*n*-pentane/acetone 10:1) to afford compound S2 (22 g, 129.2 mmol, 80%) as a red solid. The characterisation was consistent with the reported data.[1]

Compound I-*e*TEMPO

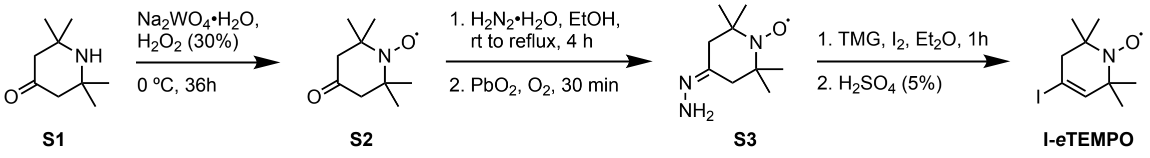

This compound was prepared following a reported procedure.[2] Briefly, Compound S2 (8.5 g, 50 mmol) dissolved in EtOH (30 mL) was added dropwise to hydrazine hydrate (64%, 0.3 mol, 15 mL) over 3 h. The reaction mixture was heated to reflux for 1 h. After cooling down, the colorless solution was evaporated to dryness, the residue was taken up in a mixture of $CHCl_3$ and MeOH (9:1, 50 mL). The organic phase was washed with brine (10 mL) and dried over anhydrous Na_2SO_4. Then PbO_2 (1.19 g, 5.0 mmol) was added and O_2 (compressed air) was bubbled through for 30 to 60 minutes. The orange solution was filtered,

evaporated and the remaining orange thick oil (crude S3) was stored at −20 °C overnight. Crude S3 was dissolved in anhydrous Et_2O (30 mL) and added dropwise to a stirred solution of I_2 (27.9 g, 0.11 mol) and tetramethyl guanidine (20.12 g, 0.175 mol) in Et_2O (50 mL). The reaction mixture was stirred at room temperature for 60 minutes, diluted with Et_2O (40 mL), and washed with water (30 mL) and with H_2SO_4 (5% in water, 60 mL). The organic phase was separated and dried over anhydrous Na_2SO_4. The solvent was removed under reduced pressure and the dark brown residue purified by column chromatography (hexane/Et_2O, 2:1) to afford compound I-*e*TEMPO (5.8 g, 20.7 mmol, 41%) as a light orange solid. The characterisation was consistent with the reported data.[2]

Compound Bpin-*e*TEMPO

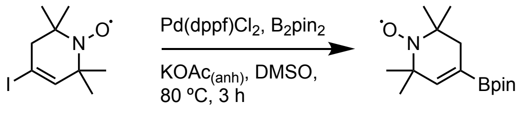

This compound was prepared following a reported procedure.[2] Briefly, a solution of bis(pinacolato)-diboron (1.10 eq., 2.49 g, 9.81 mmol), Pd(dppf)Cl_2·CH_2Cl_2 (0.056 eq., 0.36 g, 0.5 mmol), potassium acetate (3 eq., 2.63 g, 26.75 mmol) in dry DMSO (50 mL) was flushed with argon for 10 minutes. Then compound I-*e*TEMPO (1 eq., 2.5 g, 8.92 mmol) was added and the mixture was stirred at 80 °C for 3 h. After cooling down to room temperature, the mixture was poured onto water (200 mL) and extracted with Et_2O (2×200 mL). The organic phase was dried (Na_2SO_4), filtered, and evaporated and the residue was purified by column chromatography (hexane/Et_2O, 2:1 followed by hexane/EtOAc, 2:1). No starting material was recovered (as opposed to the reported procedure) and the main impurity was the homo-coupling of the compound Bpin-eTEMPO. The pure product was isolated as a light brown solid (1.85 g, 74%). The characterisation was consistent with the reported data.[2]

Compound *para*-I-BODIPY

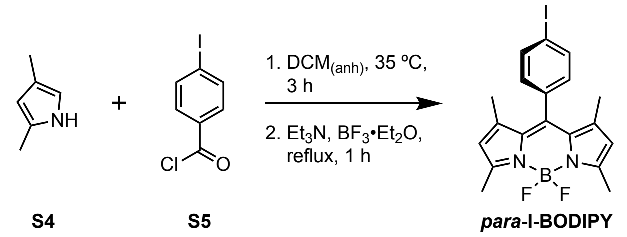

This compound was prepared following a reported procedure.[3] Briefly, 4-iodobenzoyl chloride (1 eq., 1.3 g, 4.88 mmol) and 2,4-dimethylpyrrole (2.039 eq., 0.95 g, 1.024 mL, 9.95 mmol) were dissolved in dry DCM (100 mL) and the colour of the mixture immediately changed to bright red. After refluxing for 3 h at 35 °C under inert atmosphere, the reaction mixture was cooled down to room temperature. Et_3N (4.67 eq., 2.307 g, 3.17 mL, 22.8 mmol) and boron trifluoride (5.26 eq., 3.64 g, 3.17 mL, 25.68 mmol) were added and the reaction mixture was refluxed for 30 minutes and then evaporated to dryness under reduced pressure. The residue was dissolved in chloroform (100 mL). The organic layer was washed with saturated Na_2CO_3 (100 mL) and water (2×100 mL), dried (Na_2SO_4), filtered and evaporated. The residue was purified by column chromatography on silica gel (20% chloroform/n-hexane) to yield

compound *para*-I-BODIPY (1.00 g, 46%) as an orange solid. The characterisation was consistent with the reported data.[3]

^{1}H NMR (400 MHz, $CDCl_3$) δ 7.82–7.74 (m, 2H), 7.02–6.94 (m, 2H), 5.92 (s, 2H), 2.48 (d, J = 1.2 Hz, 6H), 1.35 (s, 6H).

Compound BODIPY-*p*-*e*TEMPO

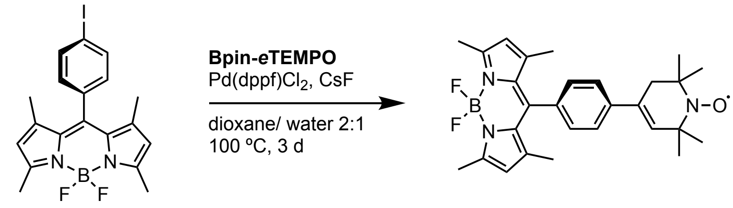

***para*-I-BODIPY** **BODIPY-*p*-*e*TEMPO**

para-I-BODIPY (1.1 eq., 16 mg, 0.036 mmol), Bpin-*e*TEMPO (1 eq., 9.055 mg, 0.032 mmol), CsF (3 eq., 14.73 mg, 0.097 mmol), and Pd(dppf)Cl_2·CH_2Cl_2 (0.05 eq., 1.32 mg, 0.0016 mmol) were dissolved in an argon saturated dioxane/water mixture (2:1, 18 mL). The reaction mixture was heated to 100 °C in a closed reaction vessel for 3 days. After cooling down to room temperature, the reaction mixture was diluted in DCM (50 mL) and extracted with brine (25 mL) and water (25 mL). The organic layer was dried (Na_2SO_4), filtered and evaporated to dryness. The residue was purified by preparative TLC on silica gel (DCM, $R_f \sim 0.2$) to yield compound BODIPY-*p*-*e*TEMPO (6.8 mg, 44%) as an orange solid.

HRMS-ESI calculated for $C_{28}H_{34}BF_2N_3O^\bullet$ $[M+H]^+$: 457.2695, found: 457.2703.

Although structural elucidation is not possible by NMR due to the presence of the radical, the spectra are reported at the end of this document.

Compound *meta*-I-BODIPY

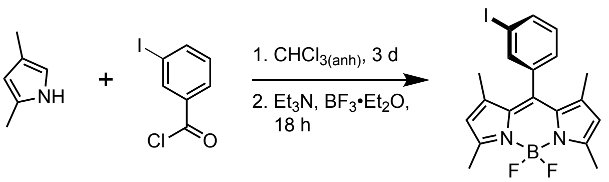

S4 **S6** ***meta*-I-BODIPY**

3-iodobenzoyl chloride (1 eq., 3.501 g, 13.14 mmol) was dissolved in dry $CHCl_3$ (140 mL), 2,4-dimethyl-pyrrole (2 eq., 2.5 g, 2.706 mL, 26.28 mmol) was added under argon and the reaction mixture was stirred at room temperature for 3 days. The reaction mixture was cooled with an ice bath and Et_3N (5.39 eq., 7.17 g, 9.85 mL, 70.86 mmol) and boron trifluoride (8.10 eq., 15.11 g, 13.14 mL, 106.47 mmol) were added dropwise. The reaction was left to react overnight at room temperature. The solvent was evaporated under reduced pressure and the residue purified by column chromatography on silica gel (3:1 hexane/chloroform). A second purification by column chromatography was necessary to obtain the pure product as an orange solid (0.8 mg, 14%). The characterisation was consistent with the reported data.[4]

^{1}H NMR (400 MHz, $CDCl_3$) δ 7.83 (dt, J = 7.7, 1.6 Hz, 1H), 7.68 (t, J = 1.7 Hz, 1H), 7.28 (dt, J = 7.7, 1.5 Hz, 1H), 7.24 (t, J = 7.6 Hz, 1H), 5.99 (s, 2H), 2.55 (d, J = 1.3 Hz, 6H), 1.43 (s, 6H).

^{13}C NMR (101 MHz, $CDCl_3$) δ 156.42, 143.42, 138.51, 137.54, 137.29, 131.19, 127.83, 121.95, 121.92, 94.83, 77.79, 77.48, 77.16, 15.17, 15.09.

^{11}B NMR (128 MHz, $CDCl_3$) δ 0.73 (t, J = 33.0 Hz).

^{19}F NMR (376 MHz, $CDCl_3$) δ −146.29 (ddd, J = 66.1, 32.8, 18.6 Hz).

Compound BODIPY-*m*-*e*TEMPO

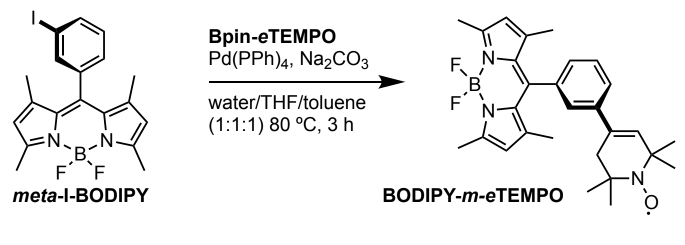

In a 50 mL round-bottom flask, *meta*-I-BODIPY (1 eq., 19.2 mg, 0.043 mmol), Bpin-*e*TEMPO (1.67 eq., 20 mg, 0.071 mmol) and sodium carbonate (3.14 eq., 14.2 mg, 0.13 mmol) were dissolved in 18 mL of water/THF/toluene (1:1:1). $Pd(PPh_3)_4$ (0.0609 eq., 3 mg, 0.0026 mmol) was added and the reaction mixture was stirred at 80 °C for 3 h (monitoring by UPLC). After reaction completion, a saturated solution of Na_2CO_3 (15 mL) was added and the mixture was extracted with dichloromethane (3×50 mL). The combined organic extracts were dried over Na_2SO_4 and filtered. The solvent was removed under reduced pressure and the residue was purified by PTLC (SiO_2, DCM, $R_f \sim 0.2$) to yield BODIPY-*m*-*e*TEMPO (10.5 mg, 51.7%) as an orange solid.

HRMS-ESI calculated for $C_{28}H_{34}BF_2N_3O^{\bullet}$ $[M+H]^+$: 477.2758, found: 477.2762.

Although structural elucidation is not possible by NMR due to the presence of the radical, the spectra are reported at the end of this document.

Compound S8

This compound was prepared following an adapted literature procedure.[5] 1,4-dibromo-2,5-dimethylbenzene (1 eq., 2 g, 7.58 mmol) and iPrO-Bpin (3-isopropoxycarbonylphenylboronic acid, 2.3 eq., 3.24 g, 3.56 mL, 17.43 mmol) were dissolved in THF (160 mL). The solution was cooled to −78 °C and a solution of *n*-BuLi 2.5 M in *n*-hexane, 6.82 mL, 17.05 mmol) was added dropwise with stirring over a period of 30 minutes. Stirring was continued at −78 °C for 1 h, the mixture was warmed to room temperature, and stirred for an additional hour. After the addition of aqueous $NaHCO_3$ and extraction with Et_2O (3×200 mL), the yellow extract was washed with brine (100 mL), and dried over Na_2SO_4. After filtration, the filtrate was evaporated to dryness under vacuum to give the title compound as a colorless solid (2.3 g, 98%). The characterisation was consistent with the reported data.[6]

^{1}H NMR (400 MHz, $CDCl_3$) δ 7.59 (s, 1H), 7.35 (s, 1H), 2.46 (s, 3H), 2.35 (s, 3H), 1.34 (s, 12H).

Compound S9

para-I-BODIPY (1 eq., 31.3 mg, 0.07 mmol), S8 (1.04 eq., 22.5 mg, 0.072 mmol), CsF (5.4 eq., 57 mg, 0.38 mmol), and $Pd(dppf)Cl_2{\cdot}CH_2Cl_2$ (0.05 eq., 2.84 mg, 0.0035 mmol) were dissolved in an argon saturated dioxane/water mixture (2:1, 5 mL). The reaction mixture was heated to 100 °C in a closed reaction

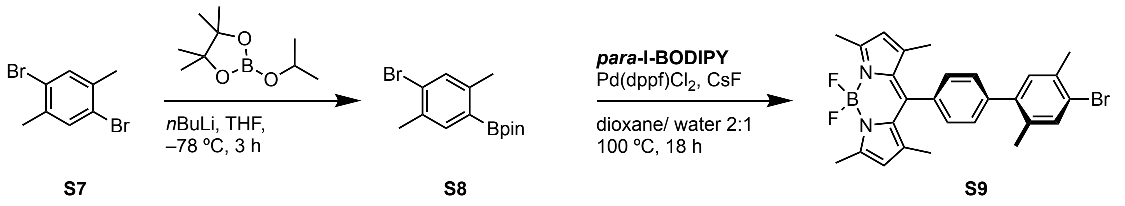

vessel for 18 h. After cooling down to room temperature, the reaction mixture was diluted in DCM (100 mL) and extracted with brine (25 mL) and water (25 mL). The organic layer was dried (Na_2SO_4), filtered and evaporated to dryness. The residue was purified by PTLC on silica gel (DCM, $R_f \sim 0.4$) to yield compound S9 (30.4 mg, 86%) as an orange solid.

^{1}H NMR (400 MHz, $CDCl_3$) δ 7.48 (s, 1H), 7.45–7.35 (m, 2H), 7.35–7.29 (m, 2H), 7.15 (s, 1H), 6.01 (s, 2H), 2.57 (s, 6H), 2.20 (s, 3H), 1.47 (s, 6H).

^{13}C NMR (101 MHz, $CDCl_3$) δ 155.69, 143.15, 141.90, 140.35, 135.36, 134.61, 134.00, 133.90, 131.94, 129.89, 128.02, 124.08, 121.44, 77.48, 77.16, 76.84, 22.44, 19.82, 14.75, 14.54.

^{11}B NMR (128 MHz, $CDCl_3$) δ 0.81 (t, J = 33.1 Hz).

^{19}F NMR (376 MHz, $CDCl_3$) δ −146.14, −146.23, −146.31, −146.40.

Compound BODIPY-xy-*e*TEMPO

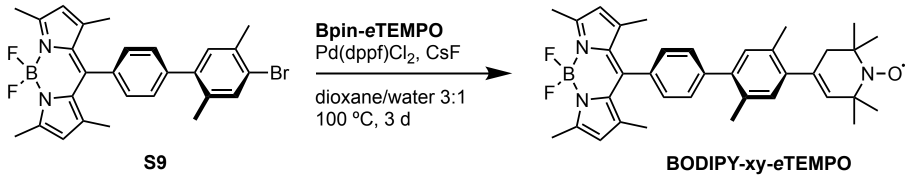

Compound S9 (1.01 eq., 10.1 mg, 0.02 mmol), Bpin-*e*TEMPO (1 eq., 5.52 mg, 0.02 mmol), CsF (3 eq., 8.98 mg, 0.059 mmol), and Pd(dppf)$Cl_2 \cdot CH_2Cl_2$ (0.05 eq., 0.805 mg, 0.001 mmol) were dissolved in an argon saturated dioxane/water mixture (3:1, 18 mL). The reaction mixture was heated to 100 °C in a closed reaction vessel for 3 days. After cooling down to room temperature, the reaction mixture was diluted in DCM (50 mL) and extracted with brine (25 mL) and water (25 mL). The organic layer was dried (Na_2SO_4), filtered and evaporated to dryness. The residue was purified by preparative TLC on silica gel (DCM, $R_f \sim 0.2$) to yield compound BODIPY-xy-*e*TEMPO (3.5 mg, 31%) as an orange solid.

HRMS-ESI calculated for $C_{36}H_{42}BF_2N_3O^{\bullet}$ $[M+H]^+$: 581.3384, found: 581.3385.

Although structural elucidation is not possible by NMR due to the presence of the radical, the spectra are reported at the end of this document.

Compound BODIPY-biph

para-I-BODIPY (1 eq., 15 mg, 0.033 mmol), Bpin-*e*TEMPO (1.1 eq., 7.48 mg, 0.037 mmol), CsF (3 eq., 15.19 mg, 0.1 mmol), and Pd(dppf)$Cl_2 \cdot CH_2Cl_2$ (0.05 eq., 1.36 mg, 1.7 μmol) were dissolved in an argon saturated dioxane/water mixture (2:1, 4 mL). The reaction mixture was heated to 100 °C in a closed reaction vessel for 18 h. After cooling down to room temperature, the reaction mixture was diluted in DCM (30 mL) and extracted with brine (15 mL) and water (15 mL). The organic layer was dried (Na_2SO_4), filtered and evaporated to dryness. The residue was purified by preparative TLC on silica gel (DCM,

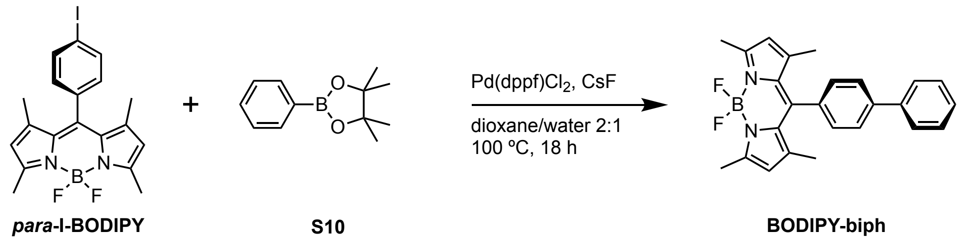

$R_f \sim 0.5$) to yield compound BODIPY-biph (6.9 mg, 52%) as an orange solid. The characterisation was consistent with the reported data.[7]

HRMS-ESI calculated for $C_{25}H_{24}BF_2N_2O^{\bullet}$ $[M+H]^+$: 401.1995, found: 401.1998.

^{1}H NMR (400 MHz, $CDCl_3$) δ 7.79–7.72 (m, 2H), 7.72–7.65 (m, 2H), 7.53–7.44 (m, 2H), 7.44–7.32 (m, 3H), 5.99 (s, 2H), 2.57 (d, J = 1.2 Hz, 6H), 1.45 (s, 6H).

^{13}C NMR (101 MHz, $CDCl_3$) δ 155.52, 143.18, 141.70, 139.99, 131.48, 128.98, 128.49, 127.88, 127.64, 127.05, 121.26, 77.35, 77.24, 77.04, 76.72, 14.58.

^{11}B NMR (128 MHz, $CDCl_3$) δ 0.80 (t, J = 33.1 Hz).

^{19}F NMR (376 MHz, $CDCl_3$) δ −146.28 (dd, J = 66.2, 32.9 Hz).

Compound ph-*e*TEMPO

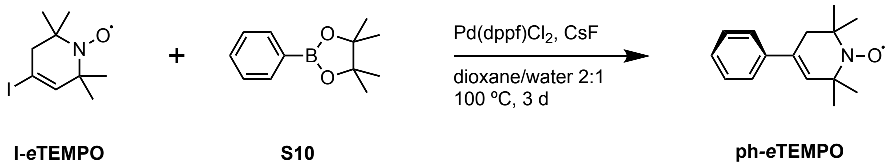

I-*e*TEMPO (1 eq., 15 mg, 0.054 mmol), 4,4,5,5-tetramethyl-2-phenyl-1,3,2-dioxaborolane (1.1 eq., 12.02 mg, 0.059 mmol), CsF (3 eq., 24.40 mg, 0.16 mmol), and $Pd(dppf)Cl_2{\cdot}CH_2Cl_2$ (0.05 eq., 2.19 mg, 2.7 μmol) were dissolved in an argon saturated dioxane/water mixture (2:1, 5 mL). The reaction mixture was heated to 100 °C in a closed reaction vessel for 3 days. After cooling down to room temperature, the reaction mixture was diluted in DCM (20 mL) and extracted with brine (15 mL) and water (15 mL). The organic layer was dried (Na_2SO_4), filtered and evaporated to dryness. The residue was purified by preparative TLC on silica gel (DCM, $R_f \sim 0.5$) to yield compound Ph-*e*TEMPO (5.2 mg, 42%) as a colorless solid.

HRMS-ESI calculated for $C_{15}H_{20}NO^{\bullet}$ $[M]^+$: 230.1539, found: 230.1539.

Although structural elucidation is not possible by NMR due to the presence of the radical, the spectra are reported at the end of this document.

2 Spectroscopic characterisation

2.1 Determination of the molar absorption coefficient of *e*TEMPO

For the calculation of the excitation energy transfer rate constants, the molar absorption coefficient of *e*TEMPO was determined in toluene solution at room temperature and compared to that of TEMPO. The data are shown in Figure S2. At the absorption maximum of 458 nm, a value of $\varepsilon = 21.2\,M^{-1}cm^{-1}$ was obtained for *e*TEMPO, while TEMPO has a molar absorption coefficient of $\varepsilon = 10.9\,M^{-1}cm^{-1}$ at 471 nm.

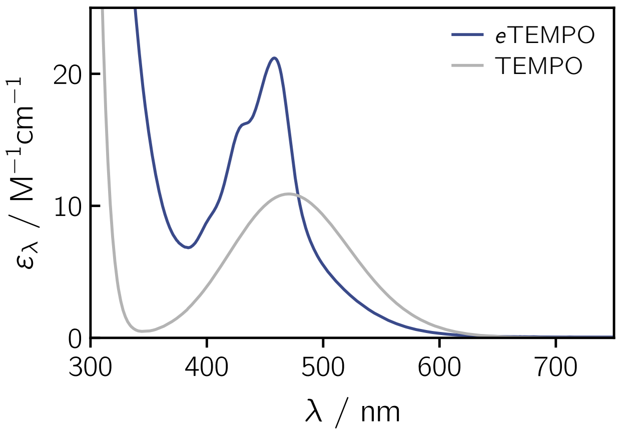

Figure S2: Determination of the molar absorption coefficient of Bpin−*e*TEMPO by UV-vis spectroscopy in toluene at room temperature.

2.2 Determination of the fluorescence lifetimes

The fluorescence lifetimes of the BODIPY−*e*TEMPO dyads and the BODIPY-biph reference were measured by single photon timing in toluene solutions at room temperature as detailed in the main text. The fluorescence traces of BODIPY−*m*−*e*TEMPO and BODIPY−*p*−*e*TEMPO were found to be slightly biexponential, which could be ascribed to the presence of different conformers in the sample, as also observed in reference 8. The contribution of the second time constant is, however, below 2 % in all cases. The fit parameters are summarised in Table S1.

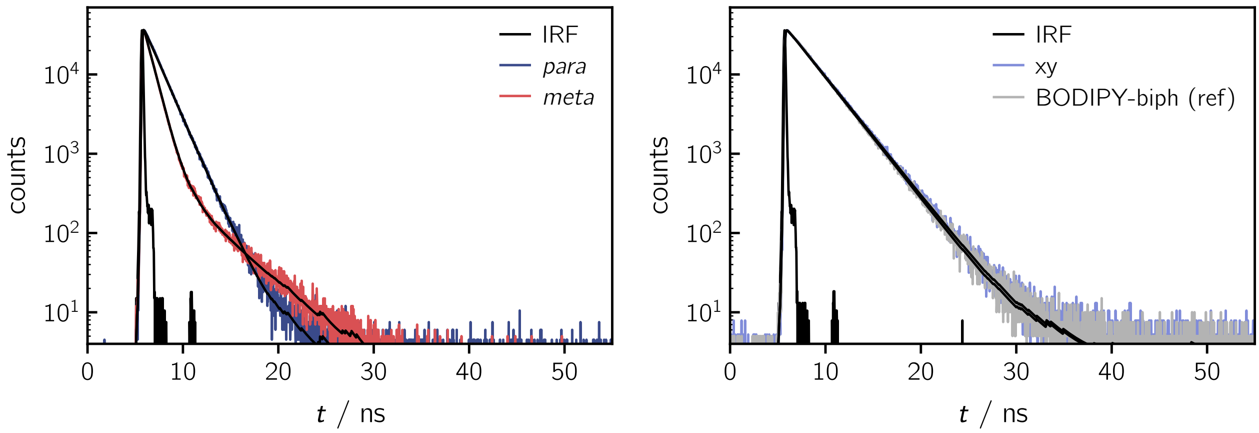

Figure S3: Determination of the fluorescence lifetimes τ_F of the BODIPY−*e*TEMPO dyads by single photon timing in toluene solution at room temperature. The data are shown together with the best mono- or biexponential fits to the data.

Table S1: Summary of the fluorescence decay constants and relative amplitudes obtained from a least-squares analysis of the SPT data in toluene.

compound	τ_1 / ns	A_1	τ_2 / ns	A_2
BODIPY−*p*−*e*TEMPO	1.54	0.995	3.68	0.005
BODIPY−*m*−*e*TEMPO	0.88	0.983	4.01	0.017
BODIPY−xy−*e*TEMPO	2.89	1.0	–	–
BODIPY−bipy (ref)	2.83	1.0	–	–

2.3 Experimental determination of the redox potentials

Cyclic voltammograms of the boronic ester of *e*TEMPO (Bpin−*e*TEMPO) and *meso*-(4-iodophenyl)-BODIPY (*para*-I-BODIPY) were recorded to determine the half-wave potentials $E_{1/2}$ of both species for an estimation of the feasibility of electron transfer from *e*TEMPO to BODIPY. The data and structures of the two molecules are shown in Figure S4. The data were recorded in *o*-dichlorobenzene with a scan speed of 100 mV/s. Initially referenced against ferrocene, the potentials were converted vs SCE (+0.47 V) for further calculations.

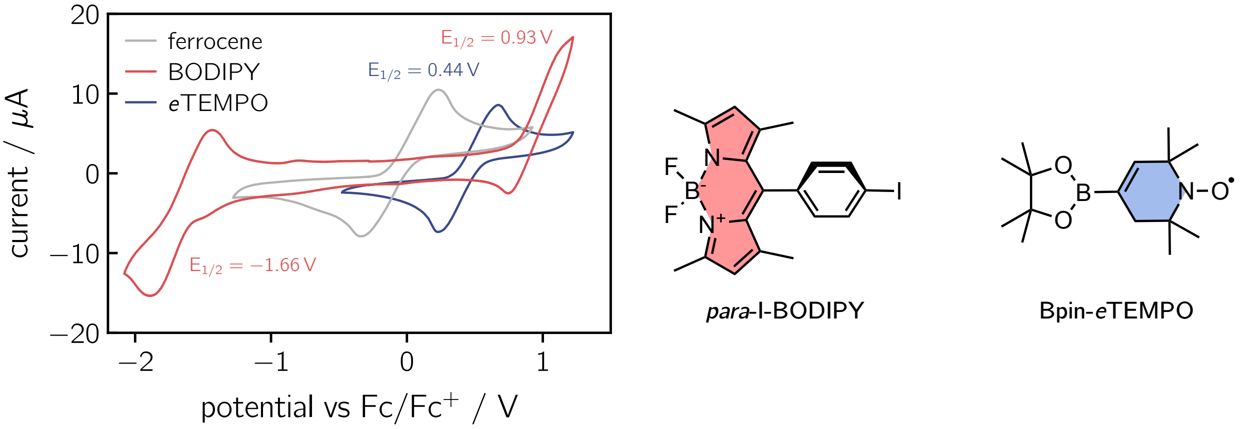

Figure S4: Determination of the redox potentials of BODIPY and *e*TEMPO (∼1 mM) by cyclic voltammetry in *o*-dichlorobenzene at room temperature. The chemical structures of the molecules used for the measurements are shown on the right.

2.4 Determination of the singlet oxygen quantum yields

Singlet oxygen quantum yields were measured as detailed in the main part. Figure S5 shows the transient singlet oxygen signal obtained for the three BODIPY−*e*TEMPO dyads and the reference compound after photoexcitation at 510 nm. A narrow spike, originating from scattered excitation light, was observed at time zero. To avoid any effects of this spike on the intensity readings used for the determination of the singlet oxygen quantum yields, only the decay beyond 2 µs was analysed. To calculate the yields given in the main part the relative intensities at 2.2 µs were used.

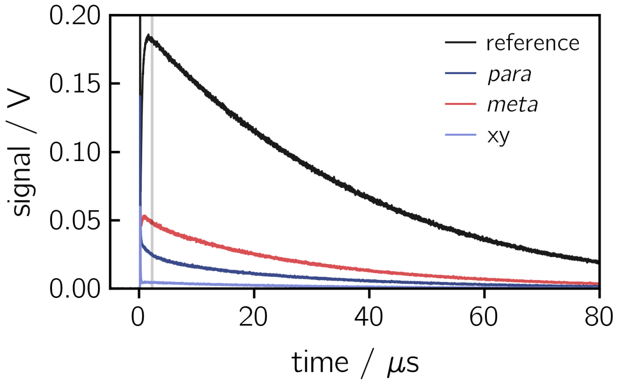

Figure S5: Measurement of the singlet oxygen signal as a function of time for the three BODIPY−*e*TEMPO dyads and the 2,6-diiodo-1,3,5,7-tetramethyl-8-phenyl-BODIPY reference compound with a known singlet oxygen quantum yield of 85 %. The grey vertical line indicates the position where the intensities were read off for the determination of the singlet oxygen quantum yields.

2.5 Calculation of the Förster energy transfer rate constant

Although the *e*TEMPO radical absorbs only weakly in the visible range, we need to consider the possibility of Förster resonance energy transfer (FRET) between the BODIPY chromophore and the *e*TEMPO radical. The molar absorption coefficient of the *e*TEMPO radical was measured by us to be $\varepsilon = 21.2\,\mathrm{M^{-1}cm^{-1}}$ at its absorption maximum in the visible range of ∼458 nm (see Figure S2). The fluorescence quantum yield of the BODIPY chromophore was measured on an absolute quantum yield spectrometer (cf. main text) and the centre-to-centre distance r_{DA} was taken from a DFT model of the BODIPY−*e*TEMPO structures (vide infra).

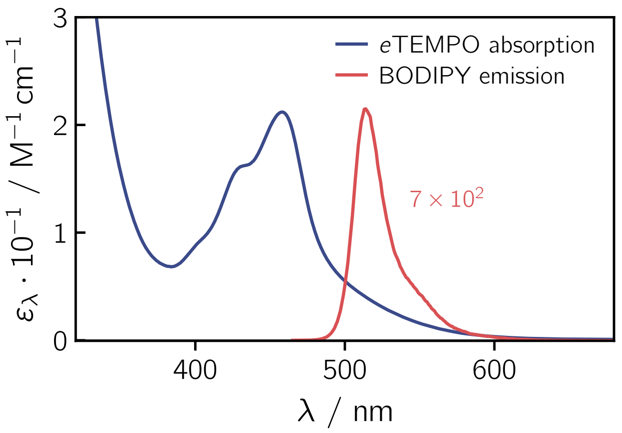

Figure S6: UV-vis absorption spectrum of the *e*TEMPO radical and illustration of the spectral overlap of fluorescence emission and radical absorption for the BODIPY-*e*TEMPO couple.

The Förster radius R_0 (obtained in nm) can be calculated from [9]

$$R_0^6 = 8.785 \cdot 10^{-11}\,\frac{\Phi_{\mathrm{F},0}^{\mathrm{D}}\,\kappa^2}{n^4}\int I_{\mathrm{F}}^{\mathrm{D}}(\lambda)\,\varepsilon^{\mathrm{A}}(\lambda)\,\lambda^4\,\mathrm{d}\lambda \tag{S1}$$

with

$$\int I_{\mathrm{F}}^{\mathrm{D}}(\lambda)\,\mathrm{d}\lambda = 1 \tag{S2}$$

where $\Phi_{\mathrm{F},0}^{\mathrm{D}}$ and $I_{\mathrm{F}}^{\mathrm{D}}$ are the fluorescence quantum yield and fluorescence intensity of the donor, ε^{A} is the molar absorption coefficient (in $\mathrm{M^{-1}cm^{-1}}$) of the acceptor and n the refractive index of the medium. The

Table S2: Overview of the results from the calculation of the FRET time constants for the three BODIPY−*e*TEMPO dyads. The following parameters were used: r_{DA} = 1.08 nm for BODIPY−*p*−*e*TEMPO, r_{DA} = 0.95 nm for BODIPY−*m*−*e*TEMPO, r_{DA} = 1.51 nm for BODIPY−xy−*e*TEMPO, $n = 1.496$ (toluene), $\tau_{F,0}^{D} = 2.83$ ns, $\Phi_{F,0}^{D} = 0.50$, $\varepsilon^{A} = 21.2\,M^{-1}cm^{-1}$, κ^2 was calculated as detailed in the main part.

compound	κ^2	τ_{FRET} / ns
BODIPY−*p*−*e*TEMPO	0.08	27
BODIPY−*m*−*e*TEMPO	0.33	3.0
BODIPY−xy−*e*TEMPO	0.10	159

orientation factor κ^2 accounts for the relative orientation of the two transition dipole moment vectors (emission of donor and absorption of acceptor) with respect to the axis connecting the FRET pair.

The energy transfer rate constant and FRET efficiency are then given as

$$\tau_{FRET}^{-1} = k_{FRET} = \frac{1}{\tau_{F,0}^{D}} \left(\frac{R_0}{r_{DA}} \right)^6 \tag{S3}$$

and

$$\Phi_{FRET} = 1 - \frac{\tau_{FRET}}{\tau_{F,0}^{D}} \tag{S4}$$

where $\tau_{F,0}^{D}$ is the fluorescence lifetime of the donor in the absence of any quenchers and r_{DA} is the centre-to-centre distance (point dipole) between donor and acceptor. The results are summarised in Table S2 and the spectral overlap between chromophore fluorescence emission and radical absorption is visualised in Figure S6.

2.6 Calculation of the driving forces for electron transfer

The calculations of the driving forces $-\Delta G_0$ for charge separation (CS) and charge recombination (CR) were performed assuming the validity of the following equations [10]

$$-\Delta G_{0,\,CS} = -\Delta G_{0,\,IP} + E_{00} \tag{S5}$$

$$-\Delta G_{0,\,CR} = \Delta G_{0,\,IP} \tag{S6}$$

$$\Delta G_{0,\,IP} = e\,[E_{ox}(D) - E_{red}(A)] + C + S \tag{S7}$$

where the subscript IP stands for ion pair, E_{00} is the energy of the first excited singlet state and $E_{ox}(D)$ and $E_{red}(A)$ are the oxidation potentials of the electron donor and reduction potentials of the electron acceptor, respectively. The terms C and S represent the coulomb and solvent correction terms, defined as

$$C = -\frac{e^2}{4\pi\varepsilon_0\varepsilon_r r_{ee}} \qquad S = \frac{e^2}{8\pi\varepsilon_0}\left(\frac{1}{r_D} + \frac{1}{r_A}\right)\left(\frac{1}{\varepsilon_r} - \frac{1}{\varepsilon_{r,\,ref}}\right) \tag{S8}$$

where r_{ee}, ε_r, ε_0, $\varepsilon_{r,\,ref}$ and r_i are the edge-to-edge distance between the reaction partners, the relative solvent permittivity, the vacuum permittivity, the relative permittivity of the solvent used to determine the redox potentials and the Van-der-Waals radii, respectively.

The edge-to-edge distance for electron transfer, as well as the Van-der-Waals radii for donor (*e*TEMPO) and acceptor (BODIPY) were estimated from DFT models. For the calculation of the Van-der-Waals radii an ellipsoidal model was assumed; the radius was calculated according to $r_{\mathrm{VdW}} = \sqrt[3]{a \cdot b \cdot c}$, where a, b, and c are the dimensions in the three different directions. The following values were obtained: $r_{\mathrm{ee}} = 8.7\,\text{Å}$ for BODIPY−*p*−*e*TEMPO, $r_{\mathrm{ee}} = 7.6\,\text{Å}$ for BODIPY−*m*−*e*TEMPO, $r_{\mathrm{ee}} = 13.0\,\text{Å}$ for BODIPY−xy−*e*TEMPO, $r_{\mathrm{D}} = 3.1\,\text{Å}$, and $r_{\mathrm{A}} = 4.8\,\text{Å}$.

The oxidation potential of *e*TEMPO in *o*-dichlorobenzene was measured to be 0.91 V vs. SCE, while for the reduction potential of BODIPY a value of −1.19 V vs. SCE was obtained (see Figure S4). The solvent *o*-dichlorobenzene has a relative dielectric constants of 9.9, while toluene has a relative dielectric constant of $\varepsilon_{\mathrm{r}} = 2.4$ at room temperature [11]. E_{00} is calculated from the crossing point of the absorption and fluorescence spectra and amounts to 2.436 eV (509 nm) for all investigated BODIPY structures. The calculated driving forces for electron transfer $-\Delta G_0$ for charge separation and charge recombination between BODIPY and *e*TEMPO are summarised in Table S3. The negative value of $-\Delta G_0$ suggests that charge separation does not occur spontaneously.

Table S3: Calculated driving forces for charge separation (CS) and charge recombination (CR) in toluene in comparison to λ.

chromophore	$-\Delta G_{0,\mathrm{CS}}$ / eV	$-\Delta G_{0,\mathrm{CR}}$ / eV	λ / eV
BODIPY−*p*−*e*TEMPO	−0.18	2.62	0.27
BODIPY−*m*−*e*TEMPO	−0.08	2.52	0.26
BODIPY−xy−*e*TEMPO	−0.41	2.85	0.29

The actual rate constants for electron transfer will further depend on the corresponding electronic matrix elements $|H_{\mathrm{AB}}|^2$ and the reorganisation energies λ. According to the classical Marcus theory for non-adiabatic electron transfer [12, 13]

$$k_{\mathrm{ET}} = A \cdot \exp\left(-\frac{(\Delta G_0 + \lambda)^2}{4\,\lambda k_{\mathrm{B}} T}\right) \tag{S9}$$

with

$$A = \frac{2\,\pi}{\hbar}\,|H_{\mathrm{AB}}|^2 \cdot \frac{1}{\sqrt{4\,\pi\lambda k_{\mathrm{B}} T}} \tag{S10}$$

where

$$\lambda = \lambda_{\mathrm{inner}} + \lambda_{\mathrm{outer}} \tag{S11}$$

and the inner and outer sphere contributions to the reorganisation energy are given as

$$\lambda_{\mathrm{inner}} = \frac{1}{2}\sum_i f_i \left(r_{\mathrm{R}}^{\mathrm{eq}} - r_{\mathrm{P}}^{\mathrm{eq}}\right)^2 \tag{S12}$$

$$\lambda_{\mathrm{outer}} = \frac{e^2}{4\pi\varepsilon_0}\left(\frac{1}{2r_{\mathrm{D}}} + \frac{1}{2r_{\mathrm{A}}} - \frac{1}{r_{\mathrm{DA}}}\right)\left(\frac{1}{n^2} - \frac{1}{\varepsilon_{\mathrm{r}}}\right) \tag{S13}$$

The outer sphere solvent reorganisation energy can be calculated using the van-der-Waals radii listed above. The inner sphere reorganisation energy for the BODIPY/*e*TEMPO couple was calculated with ORCA [14] at the DFT/B3LYP level of theory to amount to 0.2044 eV using the procedure outlined,

for instance, in reference 15 (see also Subsection 3.3 below). For all neutral or cationic species the def2-TZVP basis set was used, while the ma-def2-TZVP basis set was used for anionic species. Note, that the shifts from the use of two different basis sets cancel each other out.

The value of H_{AB} is difficult to estimate reliably, but could be responsible for a further reduction in the electron transfer rate constant. While the short distance between electron donor and acceptor could favour a large value of H_{AB}, the saturated carbon atoms of the *e*TEMPO radical will exponentially reduce the coupling between the nitroxide group and the BODIPY chromophore. In addition, an inspection of the HOMO and LUMO orbitals of the BODIPY chromophore reveals that a nodal plane runs through the axis connecting BODIPY to the *e*TEMPO substituents.

2.7 Dark state EPR spectra

Continuous wave EPR spectra were measured for all investigated BODIPY−*e*TEMPO dyads in toluene solution at room temperature to confirm the presence of the nitroxide radical. The spectra were acquired using a modulation amplitude of 1 G and a microwave power of 1 mW (20 dB). After baseline correction, the spectra were frequency-corrected to 9.75 GHz and field-corrected using a carbon fibre standard with $g = 2.002644$ [16]. The three spectra are near-identical apart from a small decrease in the intensity of the third nitrogen line in BODIPY−xy−*e*TEMPO compared to BODIPY−*m*−*e*TEMPO and BODIPY−*p*−*e*TEMPO. This intensity decrease reflects a slight increase of the rotational correlation time (*m*: 30 ps vs *p*: 45 ps vs xy: 85 ps) due to an increase in molecular size.

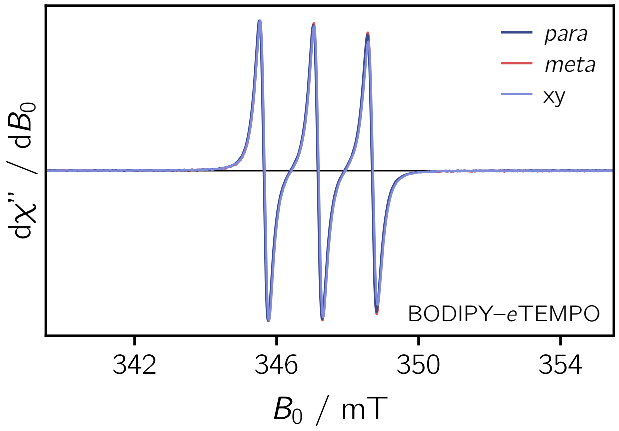

Figure S7: Normalised continuous wave EPR spectra of the three BODIPY−*e*TEMPO compounds measured at the X-band in toluene solution at room temperature.

For an accurate determination of the **g** and **A** tensors of the *e*TEMPO radical, a simultaneous fit of a field- and frequency-corrected room temperature cw EPR spectrum and a pulse Q-band EPR spectrum recorded at 80 K was performed. The data are shown in Figure S8 together with the best numerical fit using $A(^{14}N) = [16, 97]$ MHz and $g_R = [2.0103, 2.0070, 2.0025]$.

2.8 Simulations of the transient EPR spectra

To determine the magnetic parameters of the triplet excited state of the BODIPY chromophore, a trEPR spectrum of 2,6-diiodo-1,3,5,7-tetramethyl-8-phenyl-BODIPY was measured in frozen toluene solution at 80 K. The data and a numerical simulation are shown in Figure S9. The best fit, as shown in the figure, was obtained for $g_T = 2.0068$, $D_T = 2980$ MHz, and $E_T = -660$ MHz. The calculated zero-field populations are $P_{X,Y,Z} = [0.482, 0.395, 0.123]$.

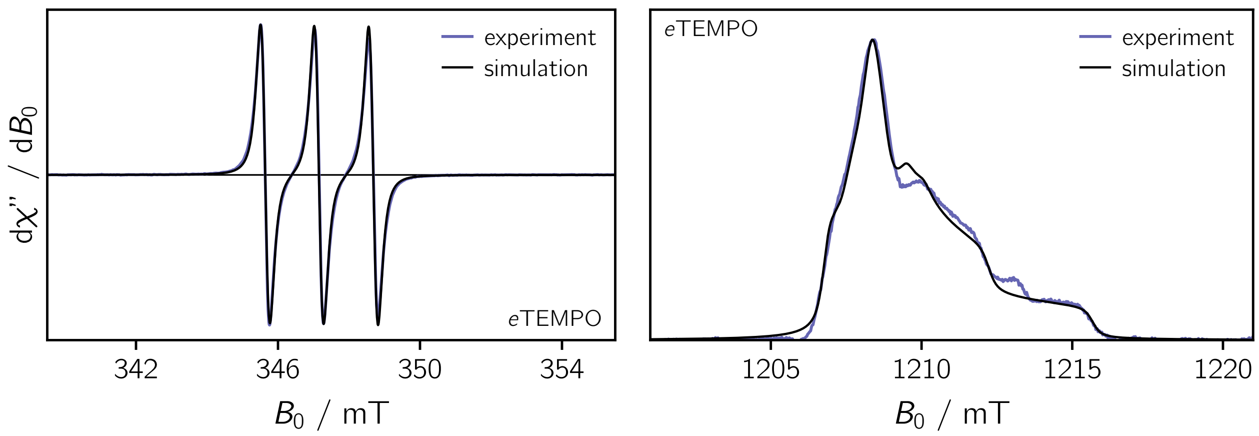

Figure S8: Determination of the magnetic parameters of *e*TEMPO by simultaneous fitting of the X-band room temperature cw EPR spectrum (*left*) and a pulse Q-band spectrum recorded in frozen toluene at 80 K (*right*).

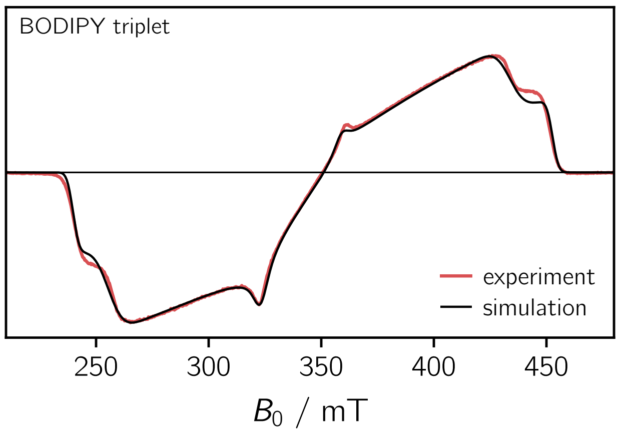

Figure S9: Transient cw EPR spectrum of 2,6-diiodo-1,3,5,7-tetramethyl-8-phenyl-BODIPY measured at the X-band in frozen toluene solution at 80 K together with the best numerical fit.

For the simulation of the coupled BODIPY−*e*TEMPO systems using the current developer version of EasySpin [17] (v6.0.0-dev.50), the g values of the triplet and doublet precursor states were kept fixed (i.e. $g_T = 2.0068$, $g_R = [2.0103, 2.0070, 2.0025]$). Only minor variations of ±10 MHz in D_T were allowed during the fitting procedure, while E_T needed to be adapted by about 100 MHz for a good agreement. A value of $E_T = -550$ MHz was found to lead to the best fit for all coupled systems. Since all spectra are well within the strong coupling regime, J_{TR} cannot be determined experimentally as its magnitude has no effect any more on the spectral shape. In the simulations, J_{TR} was therefore set to a fairly high value (+10 cm^{-1}) and kept fixed during the fitting procedure. The sign was adapted based on the exchange coupling calculations, which suggest antiferromagnetic coupling for all compounds. EasySpin uses the convention $\hat{\mathcal{H}}_J = +J\,\hat{S}_1\hat{S}_2$, meaning that a positive sign corresponds to antiferromagnetic coupling.

The only truly variable parameters were thus the linewidths and populations. The final populations obtained in the coupled triplet-radical basis (in EasySpin: Sys.initState = 'coupled') are listed in Table S4. When translating these populations into the doublet-quartet basis we obtain the populations listed in Table S5.

For reference, a direct comparison of the trEPR spectra of the three BODIPY-*e*TEMPO compounds is shown in Figure S10 together with an illustration of the spectral differences between the quartet spectra measured for the dyads and the triplet reference spectrum of 2,6-diiodo-1,3,5,7-tetramethyl-8-phenyl-BODIPY.

Table S4: Populations used for the simulation of the coupled triplet−radical systems (coupled basis). The populations are ordered from low to high energy.

	P_T (coupled basis)	P_R (coupled basis)
BODIPY−*p*−*e*TEMPO	[0.275, 0.399, 0.326]	[0.516, 0.484]
BODIPY−*m*−*e*TEMPO	[0.282, 0.386, 0.332]	[0.455, 0.545]
BODIPY−xy−*e*TEMPO	[0.314, 0.353, 0.333]	[0.493, 0.507]

Table S5: Populations after basis transformation into the doublet-quartet basis. The populations are ordered from low to high energy.

	P_Q (D_1-Q basis)	P_{D1} (D_1-Q basis)
BODIPY−*p*−*e*TEMPO	[0.187, 0.153, 0.151, 0.176]	[0.164, 0.169]
BODIPY−*m*−*e*TEMPO	[0.169, 0.161, 0.163, 0.174]	[0.167, 0.166]
BODIPY−xy−*e*TEMPO	[0.163, 0.151, 0.157, 0.196]	[0.173 0.160]

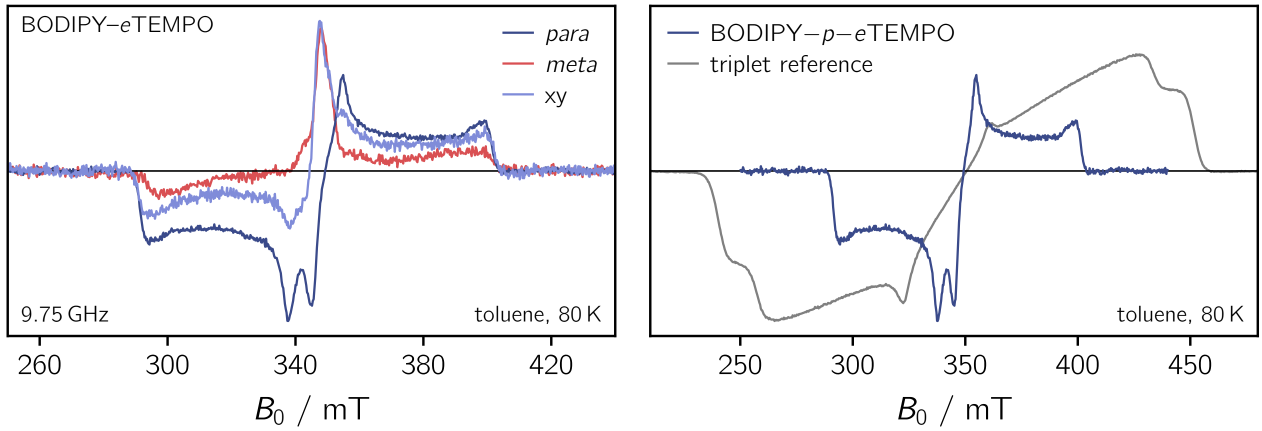

Figure S10: Direct comparison of the transient cw EPR spectra of the three BODIPY−*e*TEMPO dyads measured in frozen toluene solution at 80 K (*left*) and visual comparison of the quartet trEPR spectrum of BODIPY−*p*−*e*TEMPO and the reference triplet spectrum of 2,6-diiodo-1,3,5,7-tetramethyl-8-phenyl-BODIPY.

3 Quantum chemical calculations

All investigated structures of the BODIPY-*e*TEMPO systems were optimised at the B3LYP/def2-SVP level of theory using Gaussian 16 [18–23]. The structures of the bare BODIPY and the *e*TEMPO radical were optimised at the B3LYP/def2-TZVP level of theory using ORCA 5.0.3 [14]. All other calculations were performed using ORCA 5.0.3.

3.1 Structures and transition dipole moments

A visualisation of the optimised structures with an indication of the distances between BODIPY and *e*TEMPO is shown in Figure S11. For the determination of the transition dipole moments of BODIPY and *e*TEMPO, TD-DFT calculations were carried out at the CAM-B3LYP/def2-TZVP level of theory using the RIJCOSX approximation for the Coulomb- and exchange integrals [19, 24, 25]. The orientation of the relevant transition dipole moment within the structures is also shown in Figure S11.

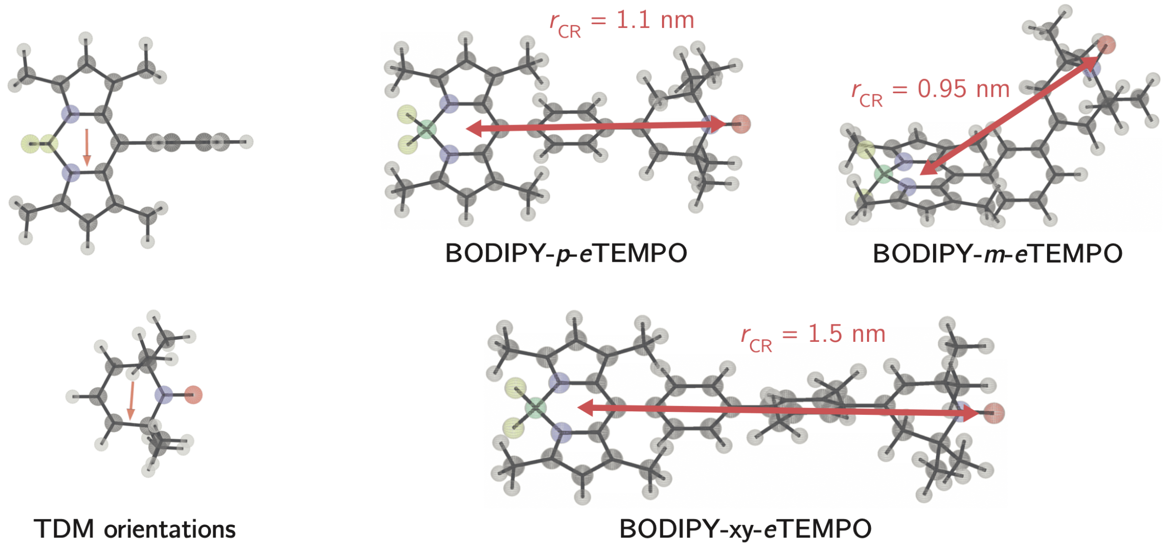

Figure S11: Transition dipole moment orientations and distances between chromophore and radical (centre of the BODIPY core to centre of N—O bond) in the BODIPY—*e*TEMPO dyads. A calculation of κ^2 based on these structures yields values of 0.08, 0.33, and 0.10 for the *para*, *meta*, and xy-linked compounds, respectively.

3.2 HOMO and LUMO orbitals of BODIPY

Figure S12 shows the HOMO and LUMO orbitals of the BODIPY chromophore, demonstrating that a nodal plane runs through the axis connecting BODIPY and *e*TEMPO.

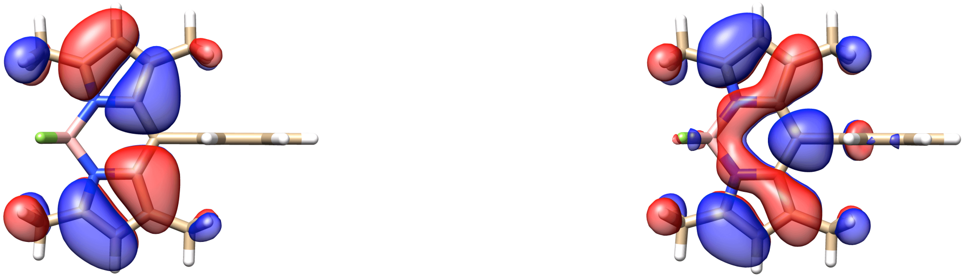

Figure S12: Visualisation of the HOMO (*left*) and LUMO (*right*) orbitals of BODIPY.

3.3 Calculation of the inner sphere reorganisation energy

The inner sphere reorganisation energy λ_{in} was calculated using

$$\lambda_{\text{in}} = 1/2(\lambda_{\text{Acc}} + \lambda_{\text{Don}}), \tag{S14}$$

where

$$\lambda_{\text{Acc}} = E^{-}_{\text{Acc}}(R^0) - E^{-}_{\text{Acc}}(R^-) + E^{0}_{\text{Acc}}(R^-) - E^{0}_{\text{Acc}}(R^0), \tag{S15}$$

$$\lambda_{\text{Don}} = E^{+}_{\text{Don}}(R^0) - E^{+}_{\text{Don}}(R^+) + E^{0}_{\text{Don}}(R^+) - E^{0}_{\text{Don}}(R^0). \tag{S16}$$

λ_{Acc} and λ_{Don} describe the contributions from the acceptor (i.e. the bare BODIPY chromophore) and the donor (*e*TEMPO radical). R^0, R^- and R^+ refer to the equilibrium structures of the corresponding neutral, anionic and cationic species. Similarly, E^0, E^- and E^+ refer to the SCF energies of the corresponding neutral, anionic and cationic species at a certain geometry. The structures were optimised and the SCF energies were calculated at the B3LYP/def2-TZVP level of theory for all neutral and cationic species, while for the anionic BODIPY chromophore the B3LYP/ma-def2-TZVP level of theory was used [26].

3.4 Exchange coupling calculations

The orbitals, that were later used as starting orbitals in the CASSCF procedure, were computed using TD-DFT at the CAM-B3LYP/def2-TZVP level of theory with the RIJCOSX approximation for the Coulomb- and exchange integrals [19, 24]. TD-DFT requires only a short computing time and therefore provides a quick overview of the orbitals that are crucial for the excited state mechanism and thus define the active space in the CASSCF procedure. As expected, the active orbitals in all investigated molecules turned out to be the chromophore HOMO and LUMO orbitals as well as the radical SOMO.

The excited state exchange interactions were calculated using QD-NEVPT2/def2-TZVP on an optimised CASSCF(3,3) chromophore triplet state wavefunction with starting orbitals obtained as described above [27]. The calculations were again accelerated by the RIJCOSX approximation. For an easier interpretation of the calculated wavefunctions, the active orbitals, which are shown in Figure S13, were localised by the Foster-Boys method [28].

The excited state exchange interaction between the chromophore triplet state and the radical doublet state can be calculated by:

$$J_{\text{TR}} = 2/3(E_{\text{D}_1} - E_{\text{Q}_0}), \tag{S17}$$

or approximately by:

$$J_{\text{TR}} = \frac{J_{12} + J_{23}}{2}, \tag{S18}$$

where D_1 is the trip-doublet state and Q_0 is the trip-quartet state. J_{12} and J_{23} are the exchange interactions between the HOMO and SOMO and between the LUMO and SOMO electrons, respectively.

The individual exchange interactions were extracted by a numerical effective Hamiltonian, that corresponds to the Heisenberg-Dirac-Van-Vleck-Hamiltonian. The exact procedure for the extraction of excited states exchange interactions was shown recently [29]. The effective Hamiltonian was calculated using:

$$H^{\text{eff}}_{IJ} = \sum_i \langle I|\varphi_i\rangle \, E_i \, \langle\varphi_i|J\rangle \,, \tag{S19}$$

where φ_i are the projected and orthonormalised wavefunctions of Q_0, D_1 and the sing-doublet state D_2.

I and J are determinants of the neutral space spanned by $|\alpha\alpha\beta\rangle$, $|\alpha\beta\alpha\rangle$, $|\beta\alpha\alpha\rangle$.

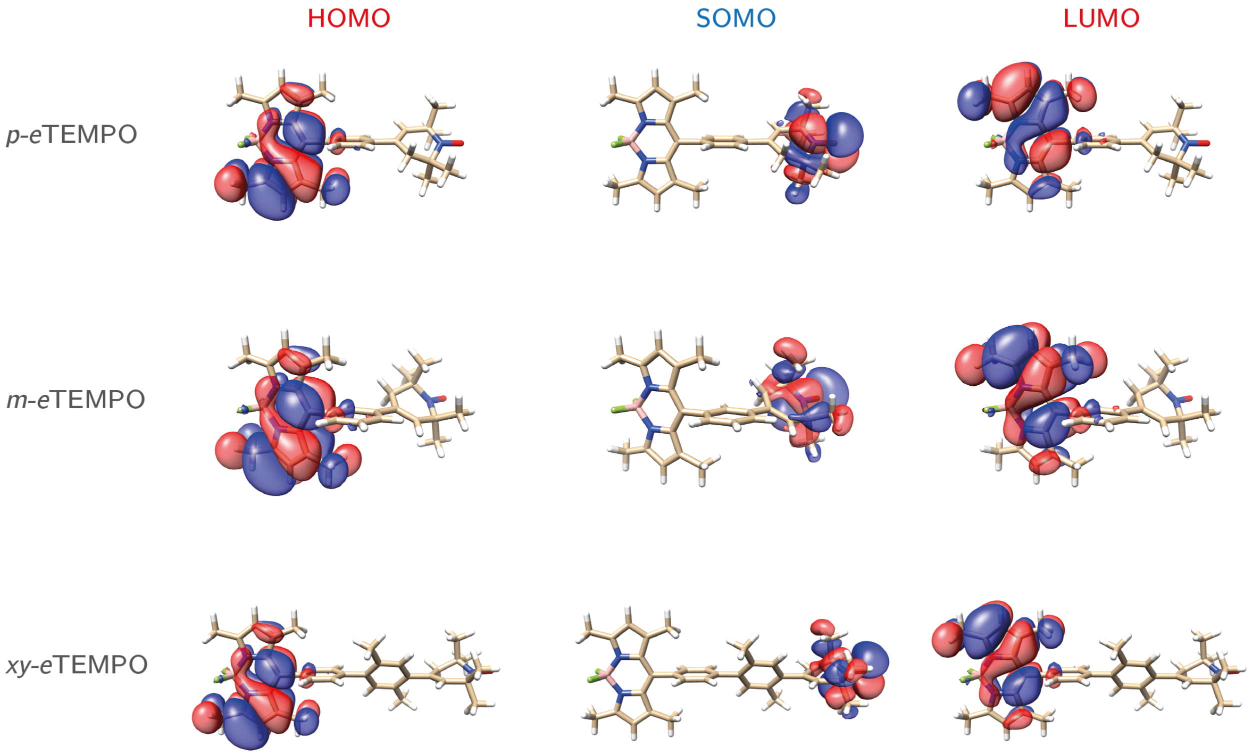

Figure S13: Localised CAS(3,3) orbitals of the investigated compounds.

4 NMR spectra

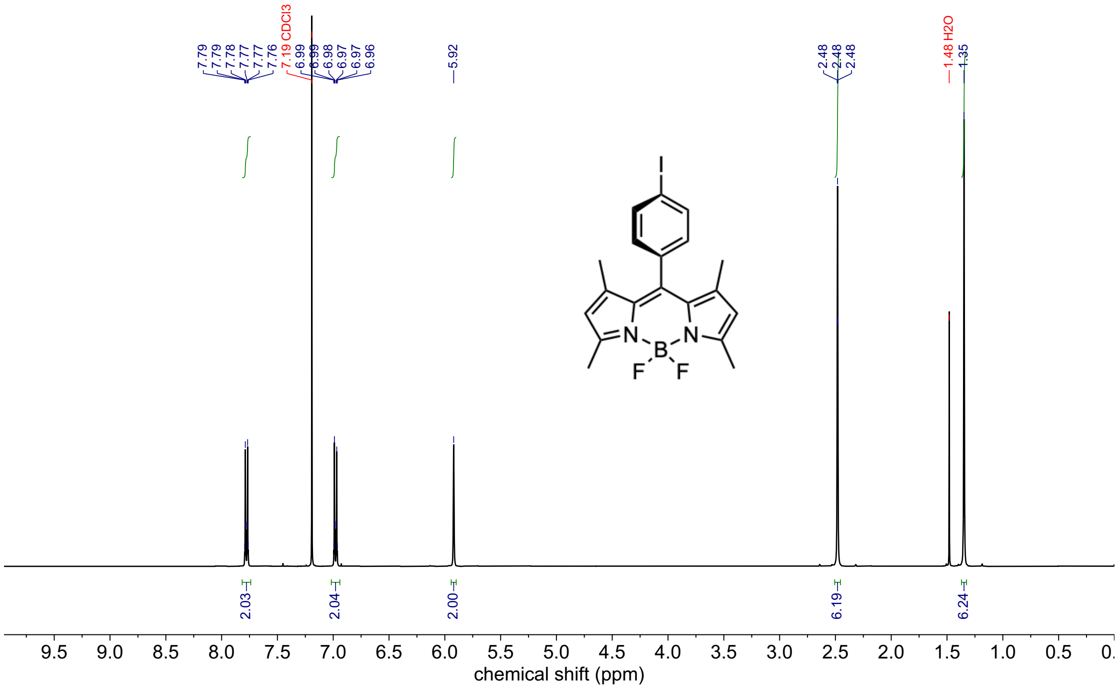

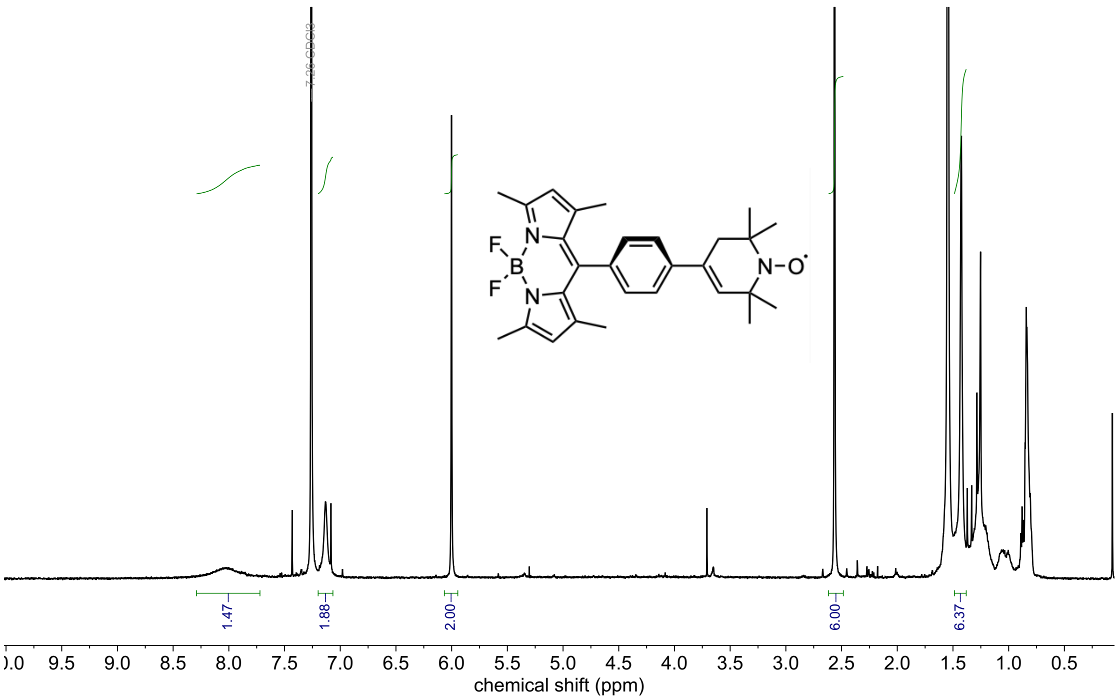

Figure S14: ^{1}H NMR of compounds *para*-I-BODIPY (*top*) and BODIPY-*p*-*e*TEMPO (*bottom*).

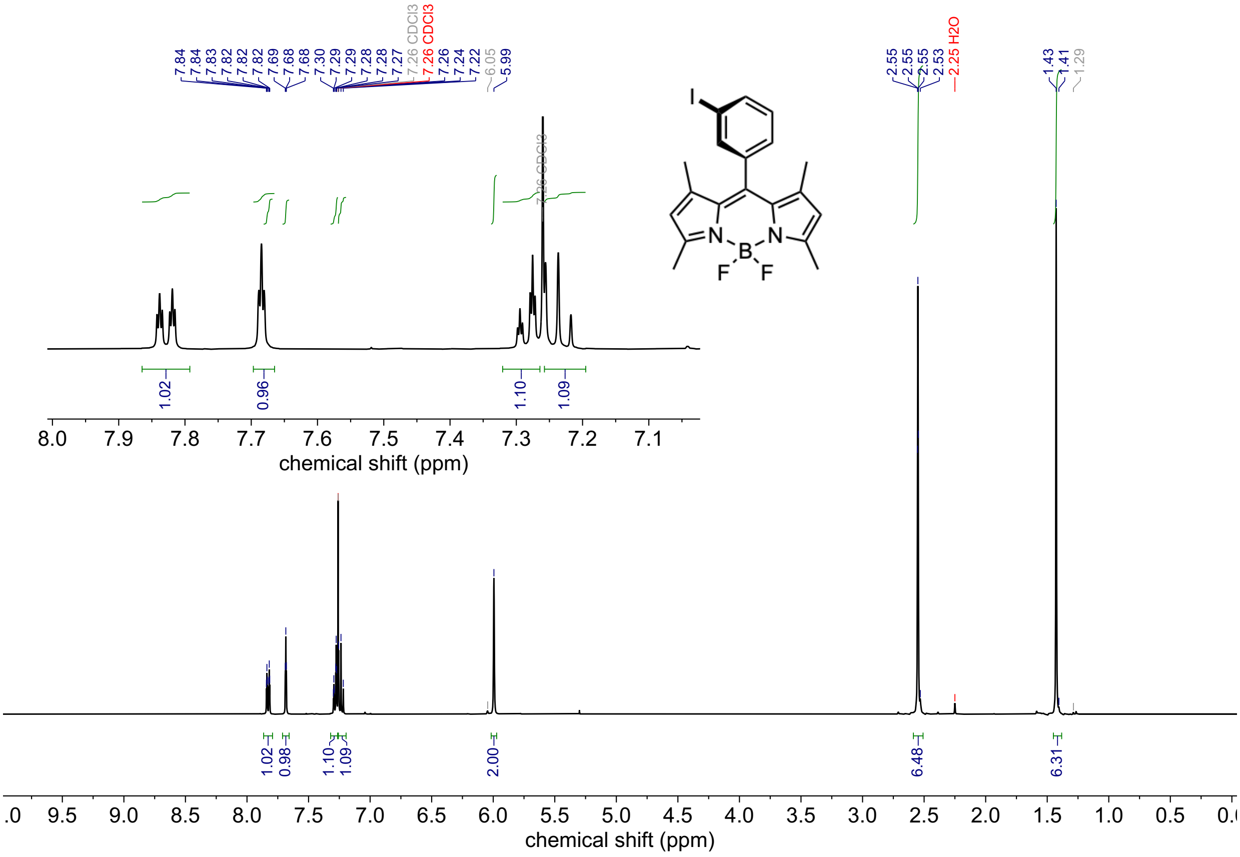

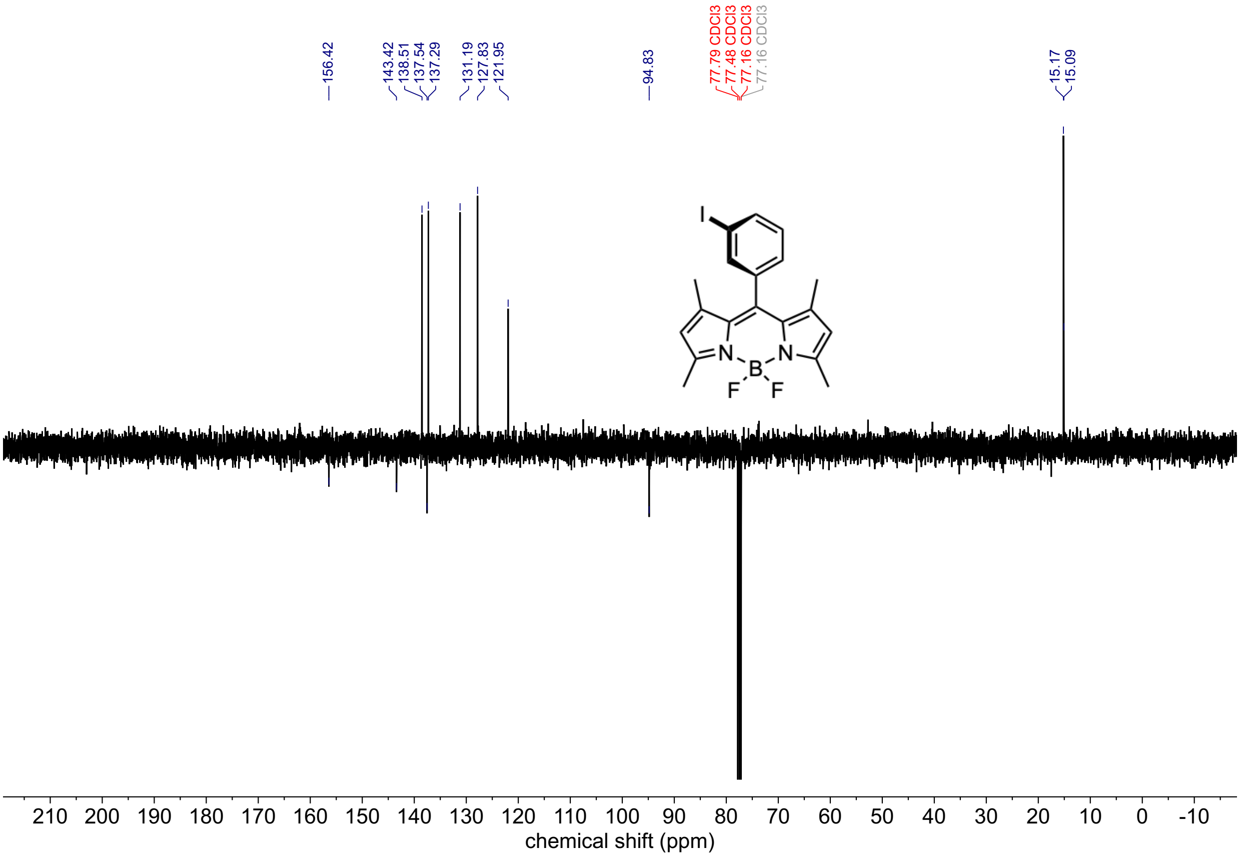

Figure S15: ^{1}H and APT (^{13}C) NMR of compound *meta*-I-BODIPY.

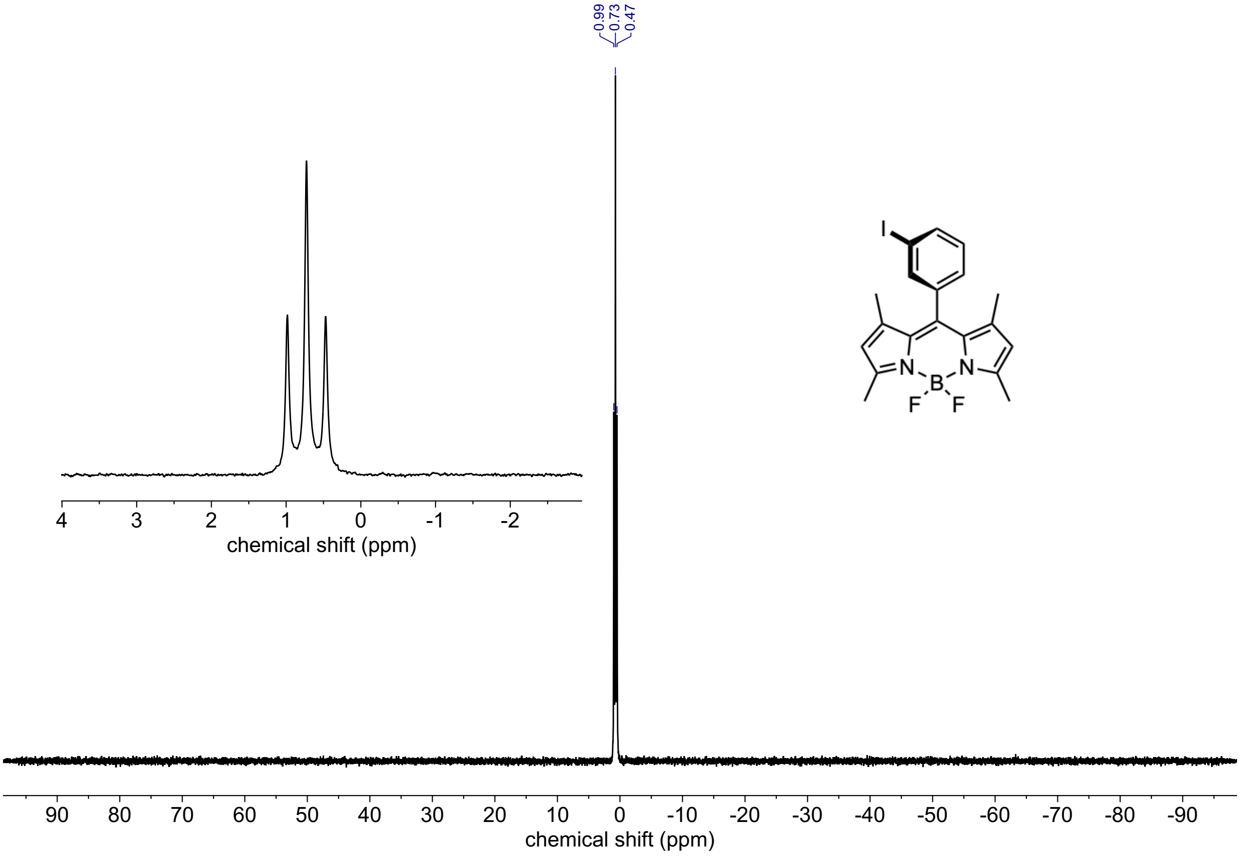

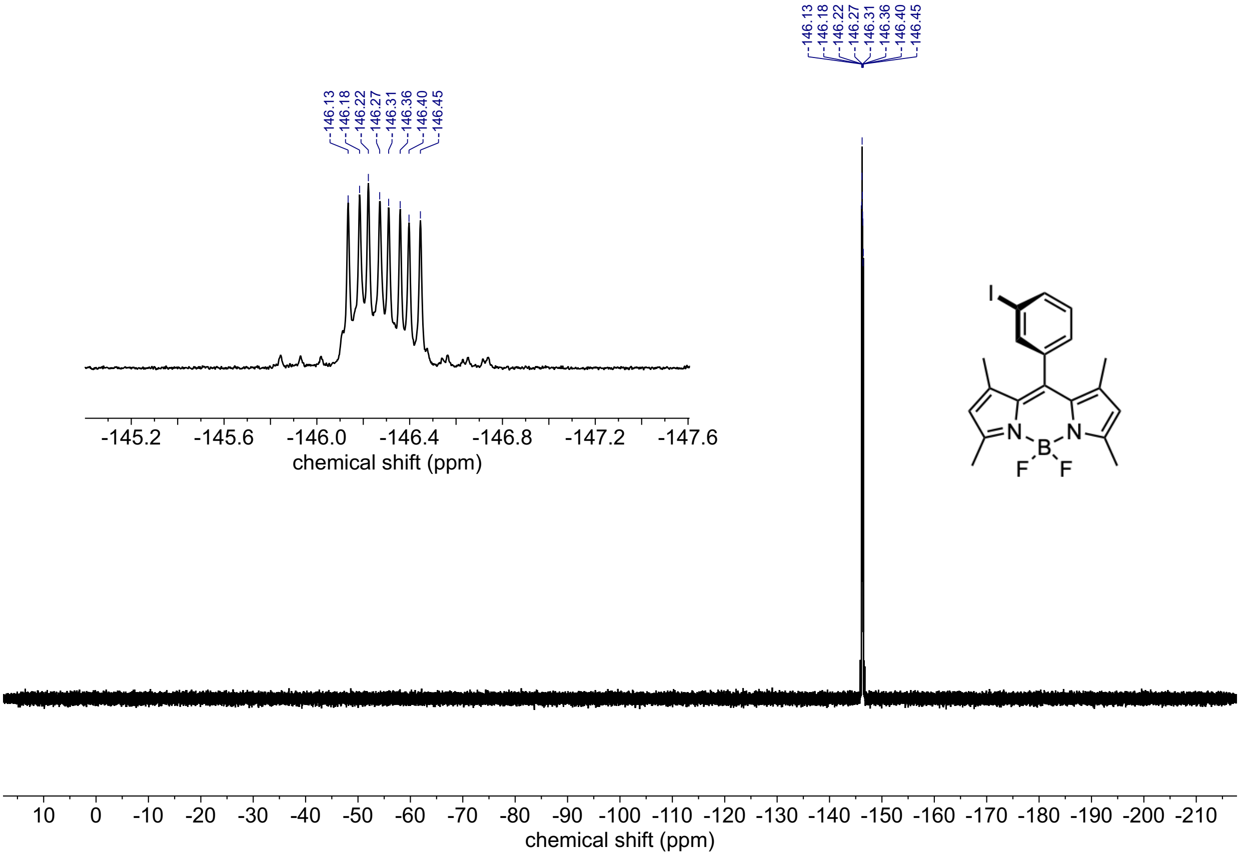

Figure S16: ^{11}B and ^{19}F NMR spectra of compound *meta*-I-BODIPY.

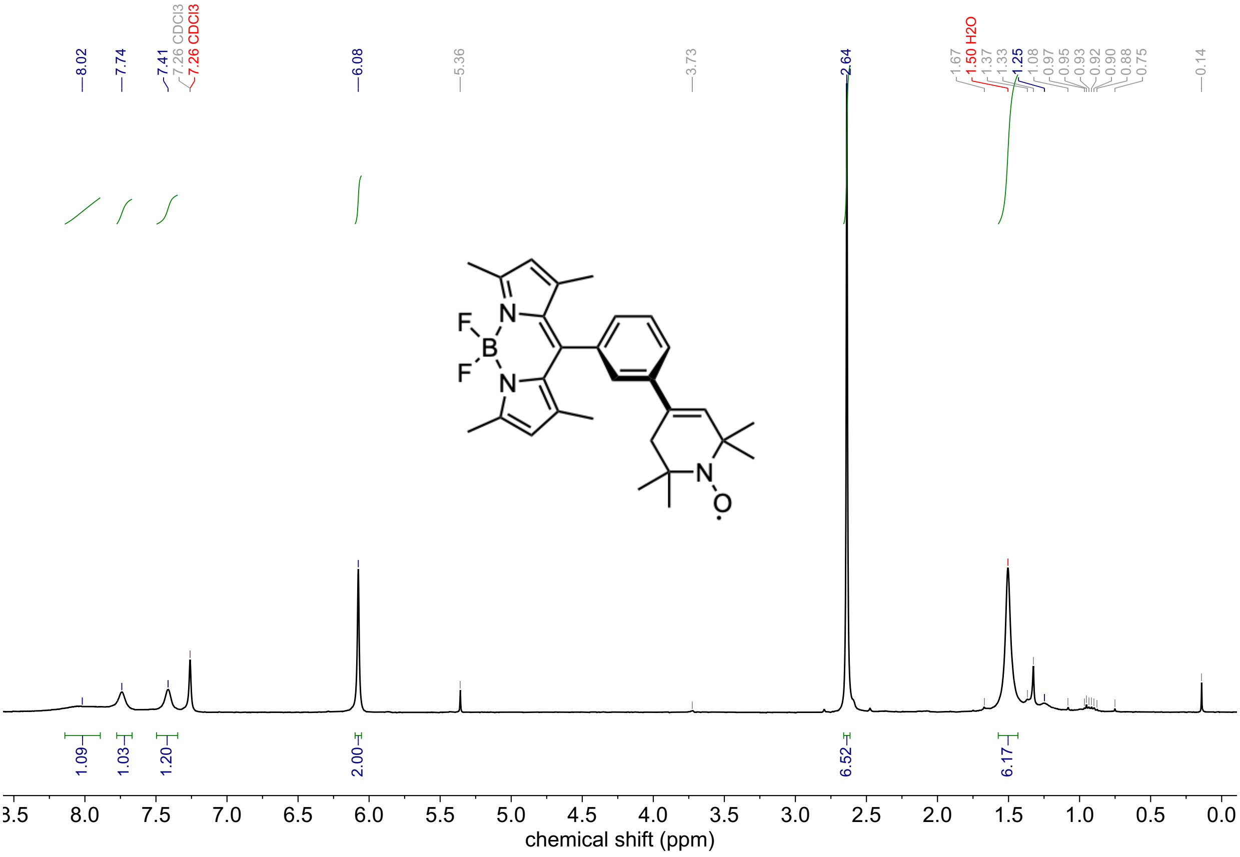

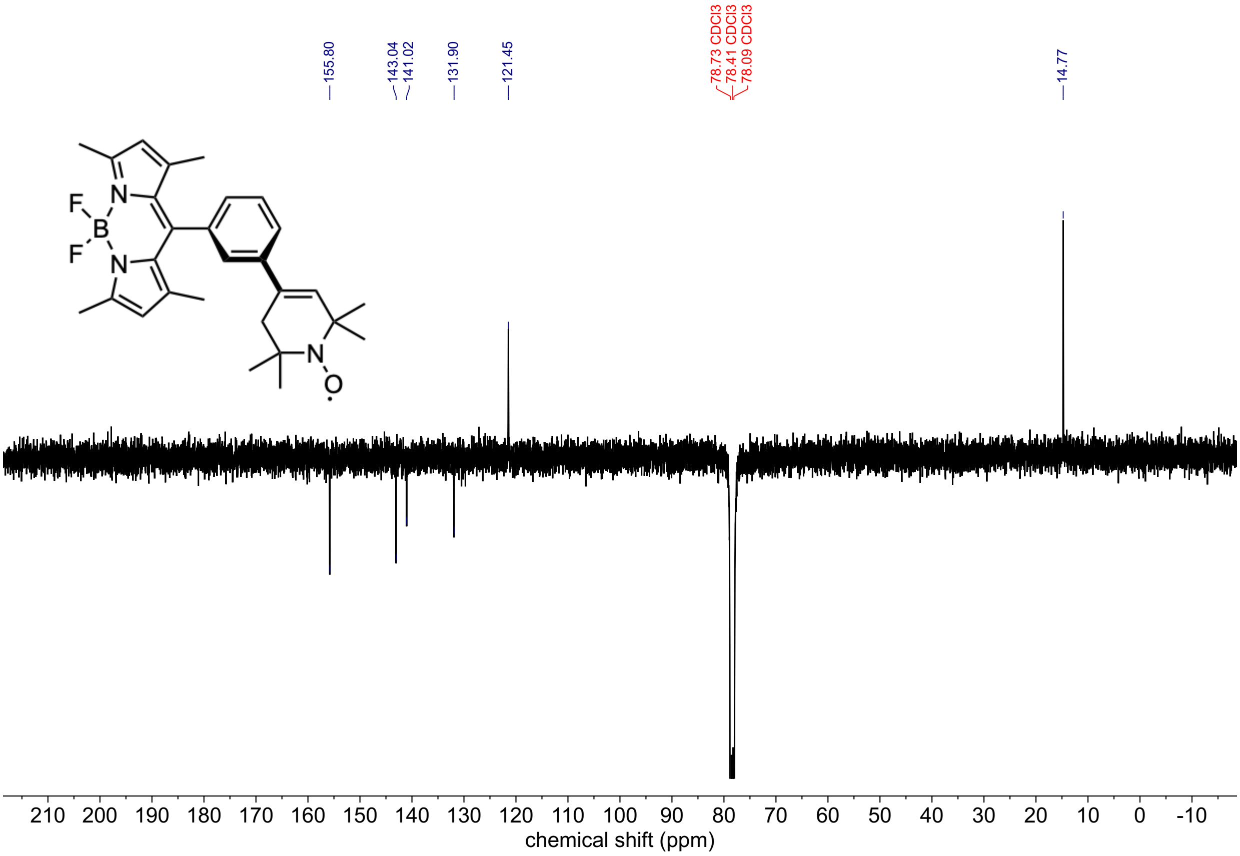

Figure S17: ^{1}H and APT (^{13}C) NMR spectra of compound BODIPY-*m*-*e*TEMPO.

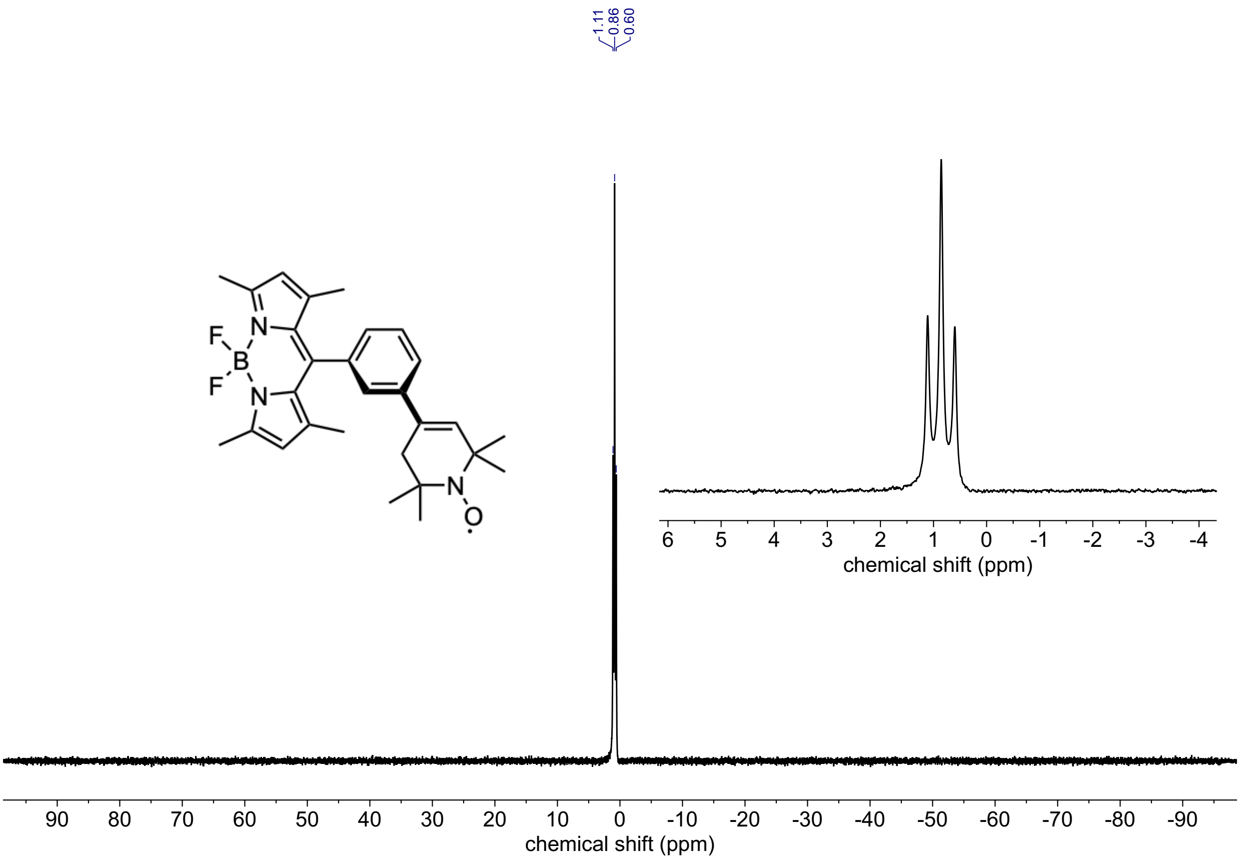

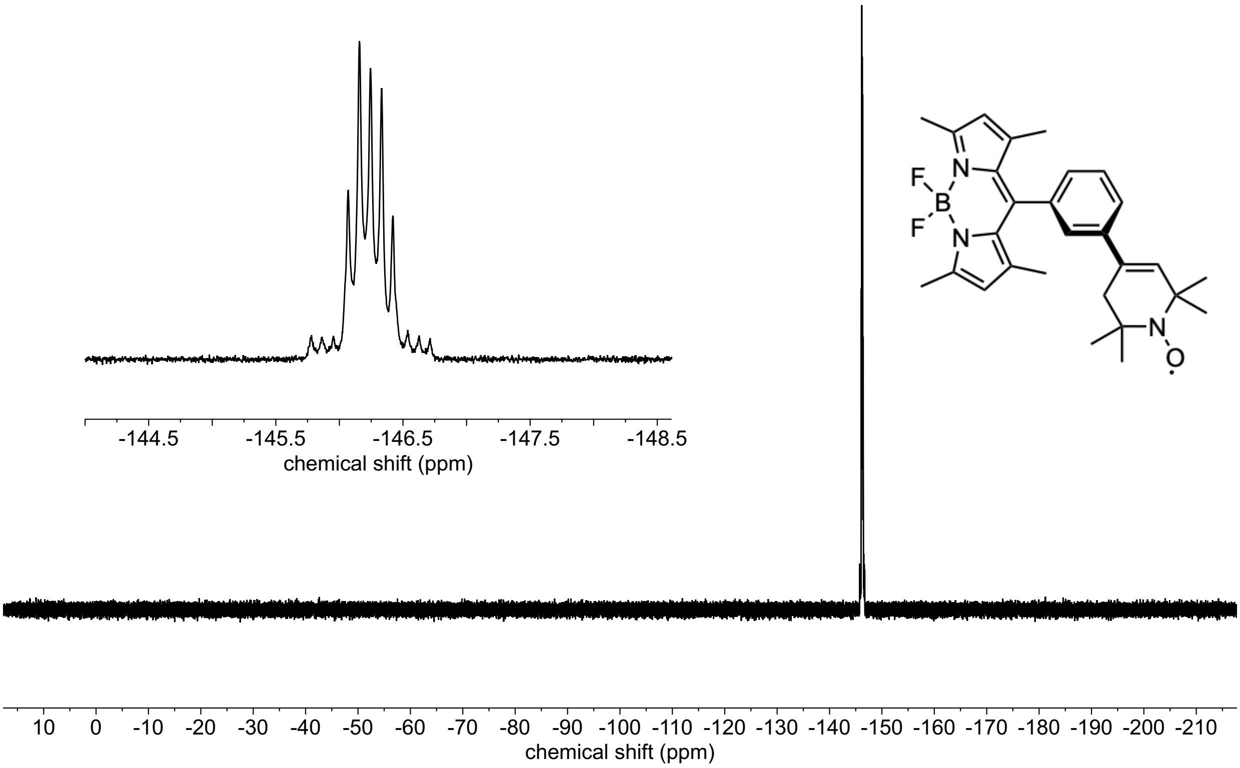

Figure S18: ^{11}B and ^{19}F NMR spectra of compound BODIPY-*m*-*e*TEMPO.

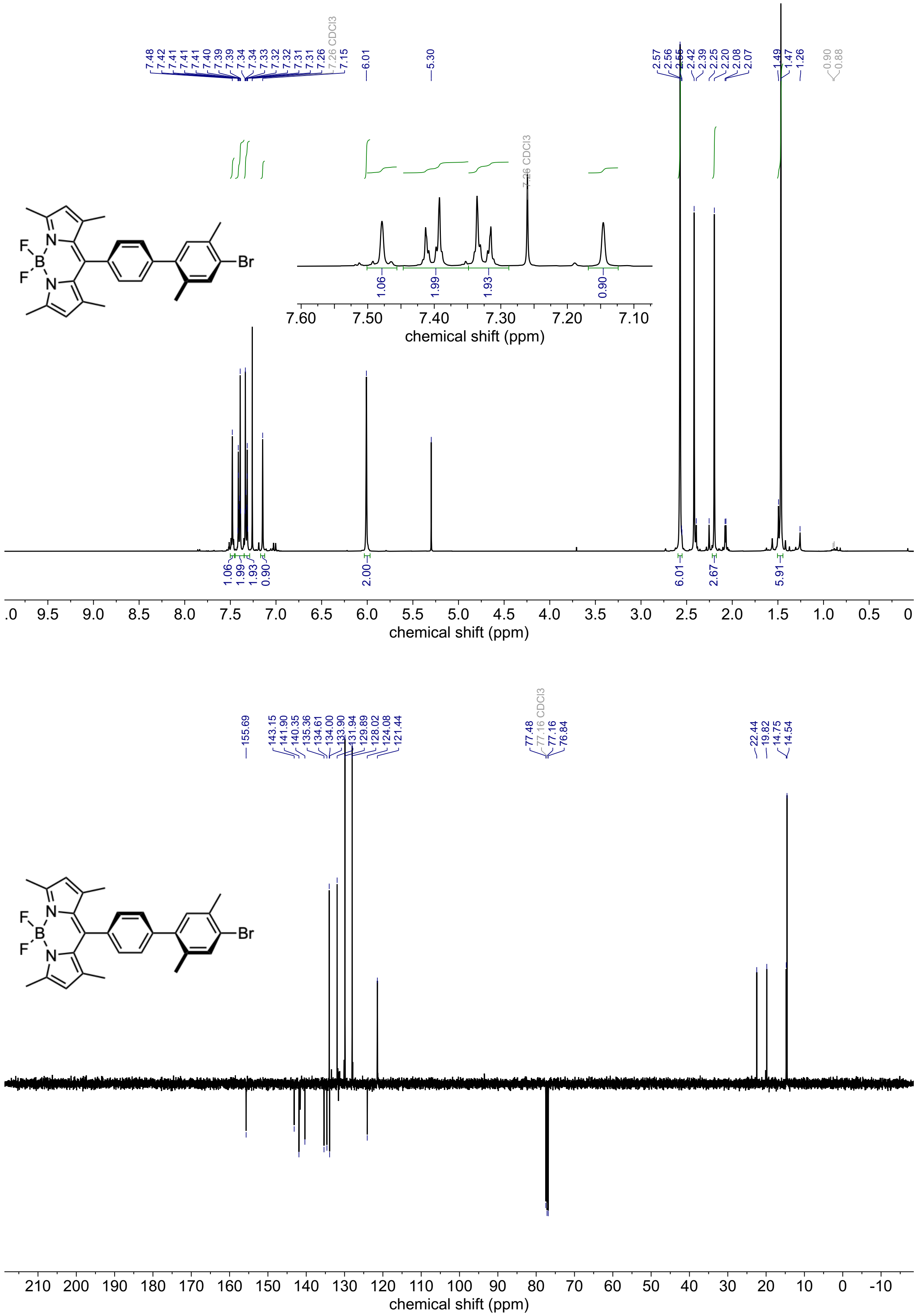

Figure S19: ^{1}H and APT (^{13}C) NMR spectra of compound S9.

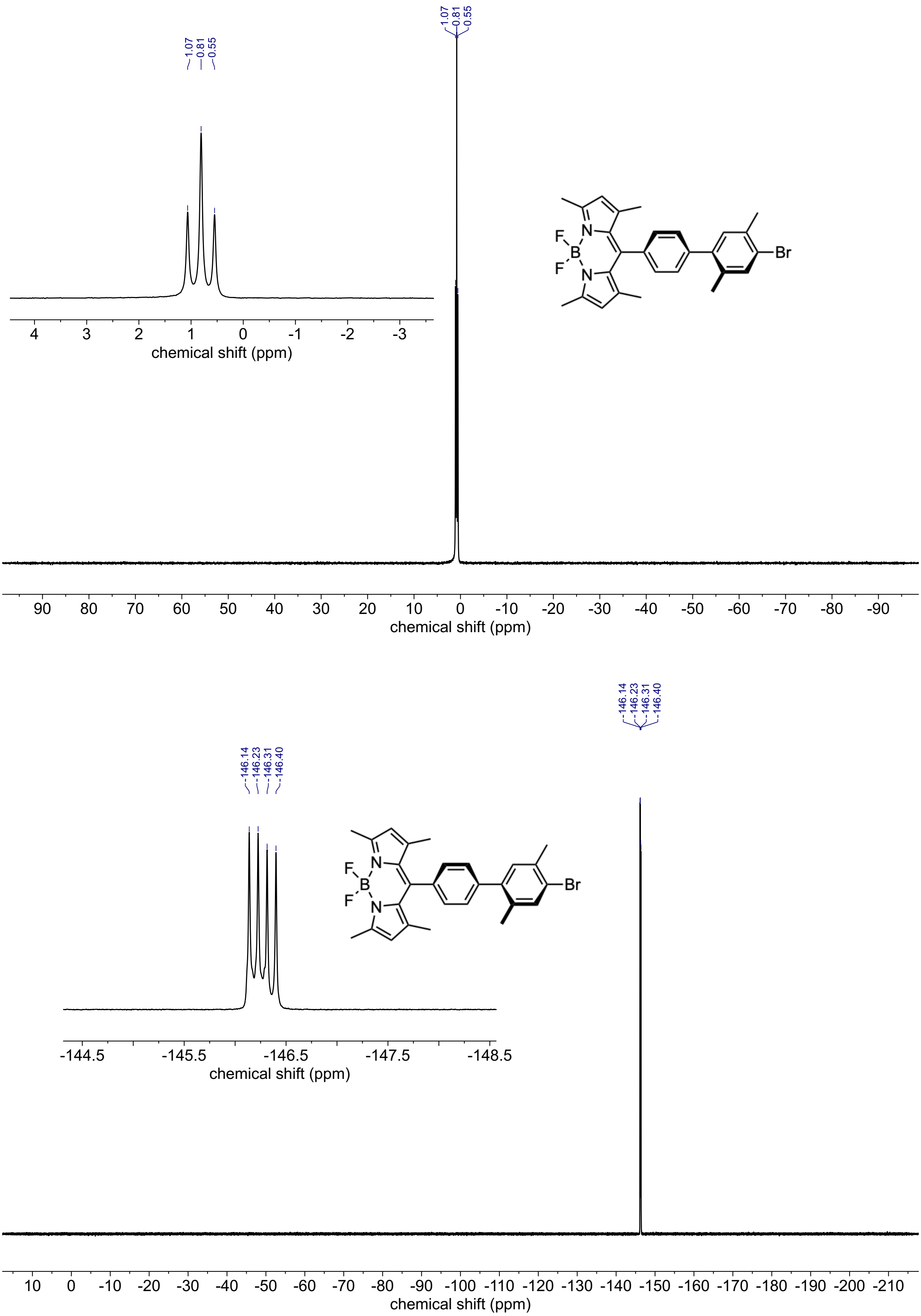

Figure S20: ^{11}B and ^{19}F NMR spectra of compound S9.

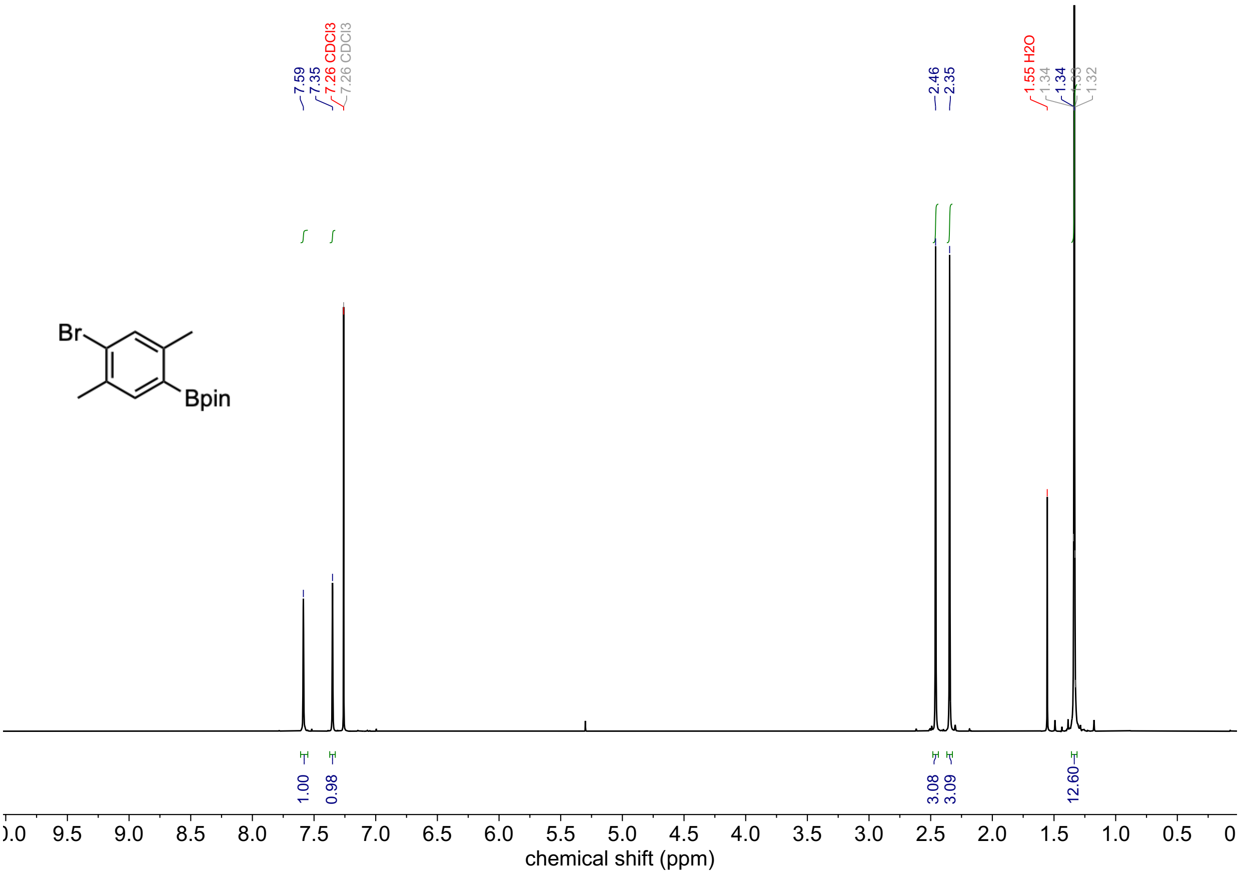

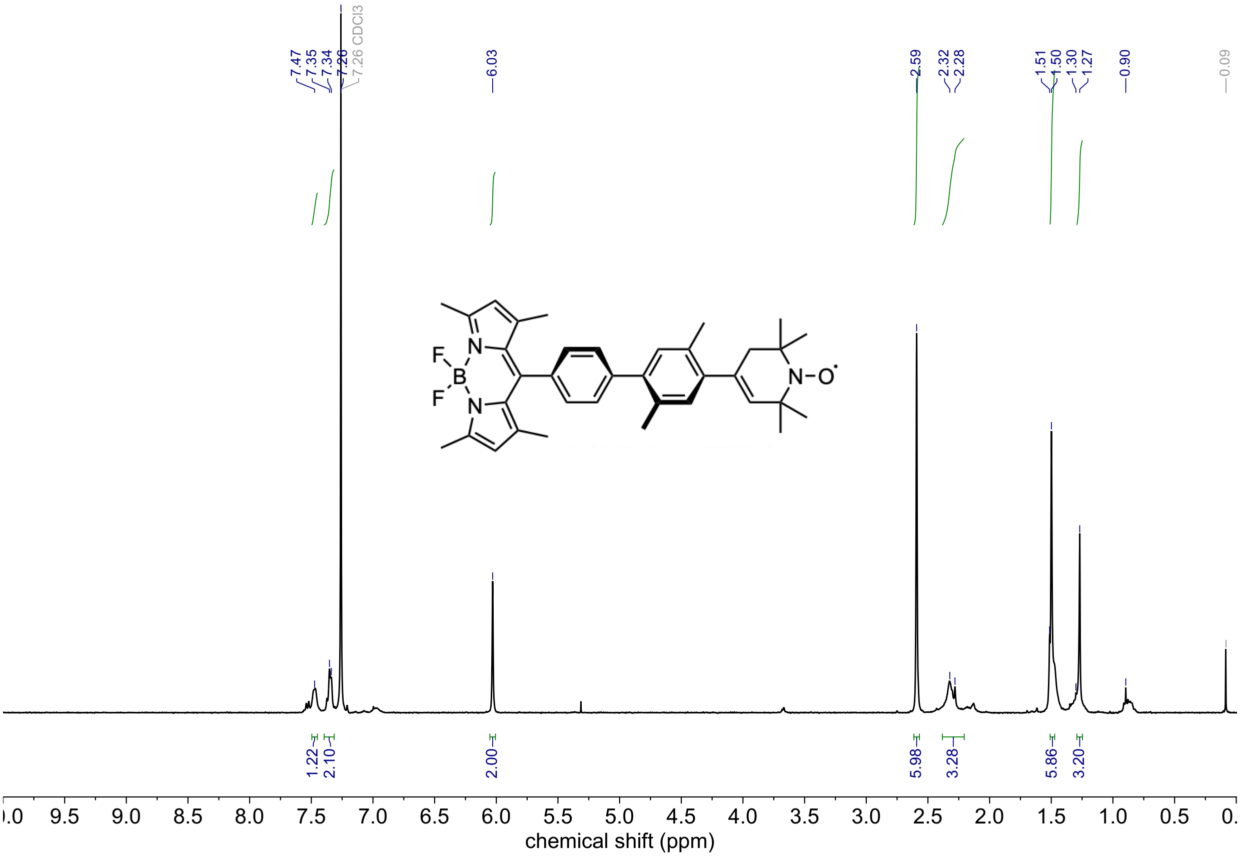

Figure S21: ^{1}H NMR spectra of compounds S8 (*top*) and BODIPY-xy-*e*TEMPO (*bottom*).

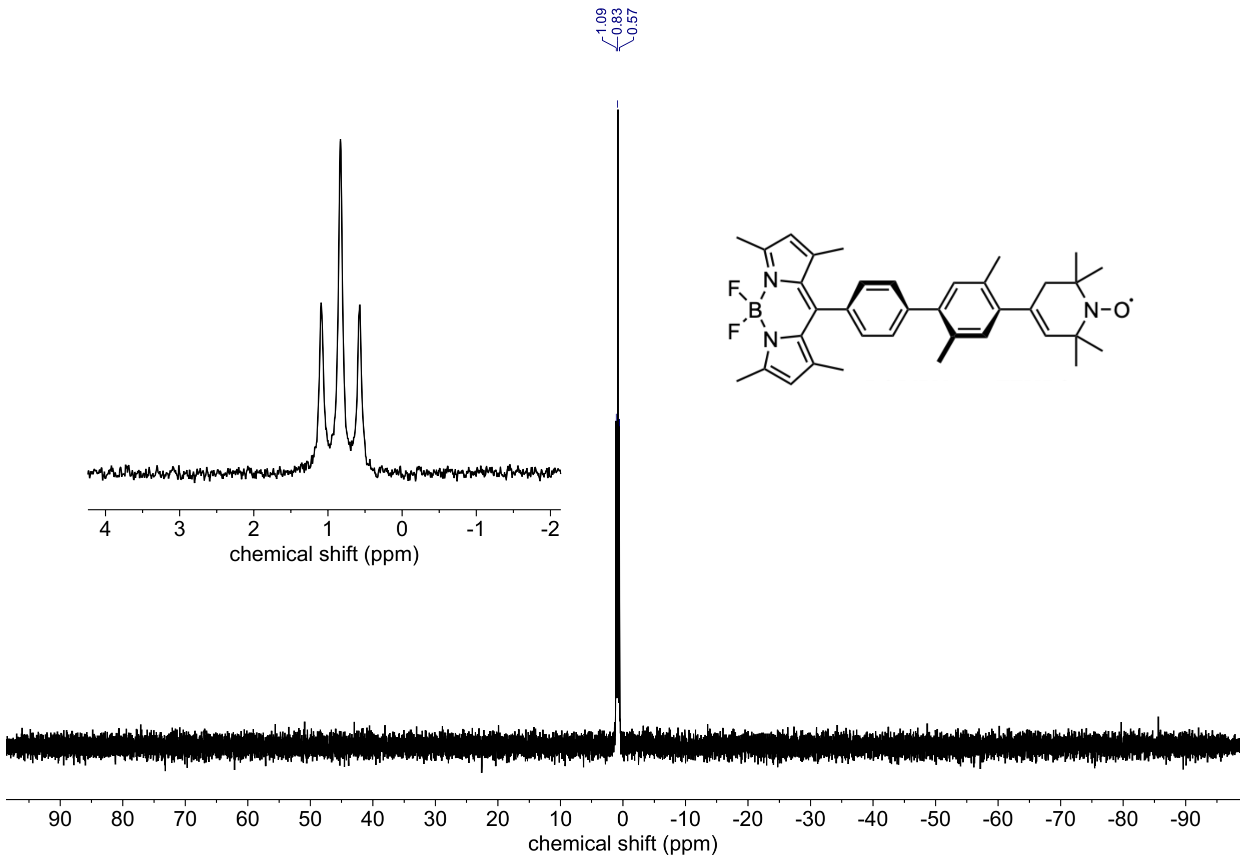

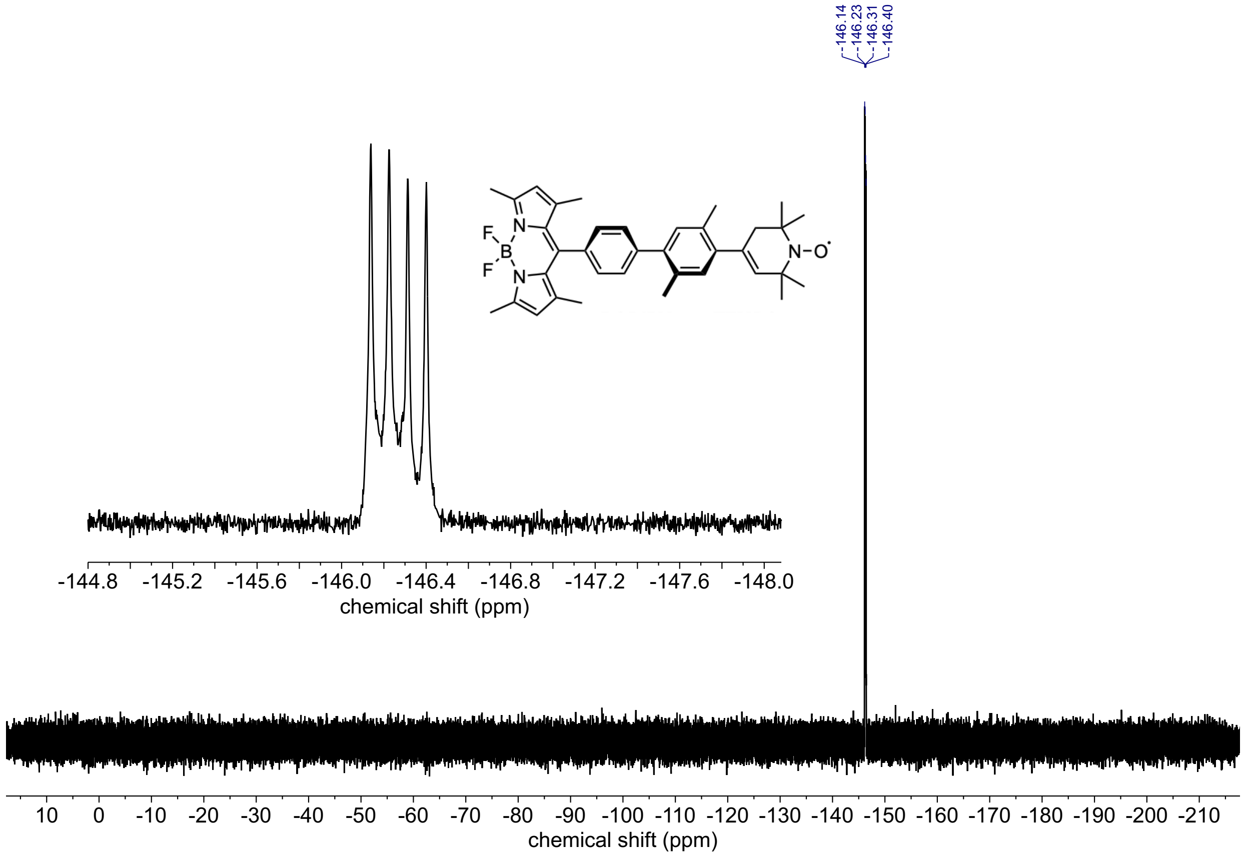

Figure S22: ^{11}B and ^{19}F NMR spectra of compound BODIPY-xy-*e*TEMPO.

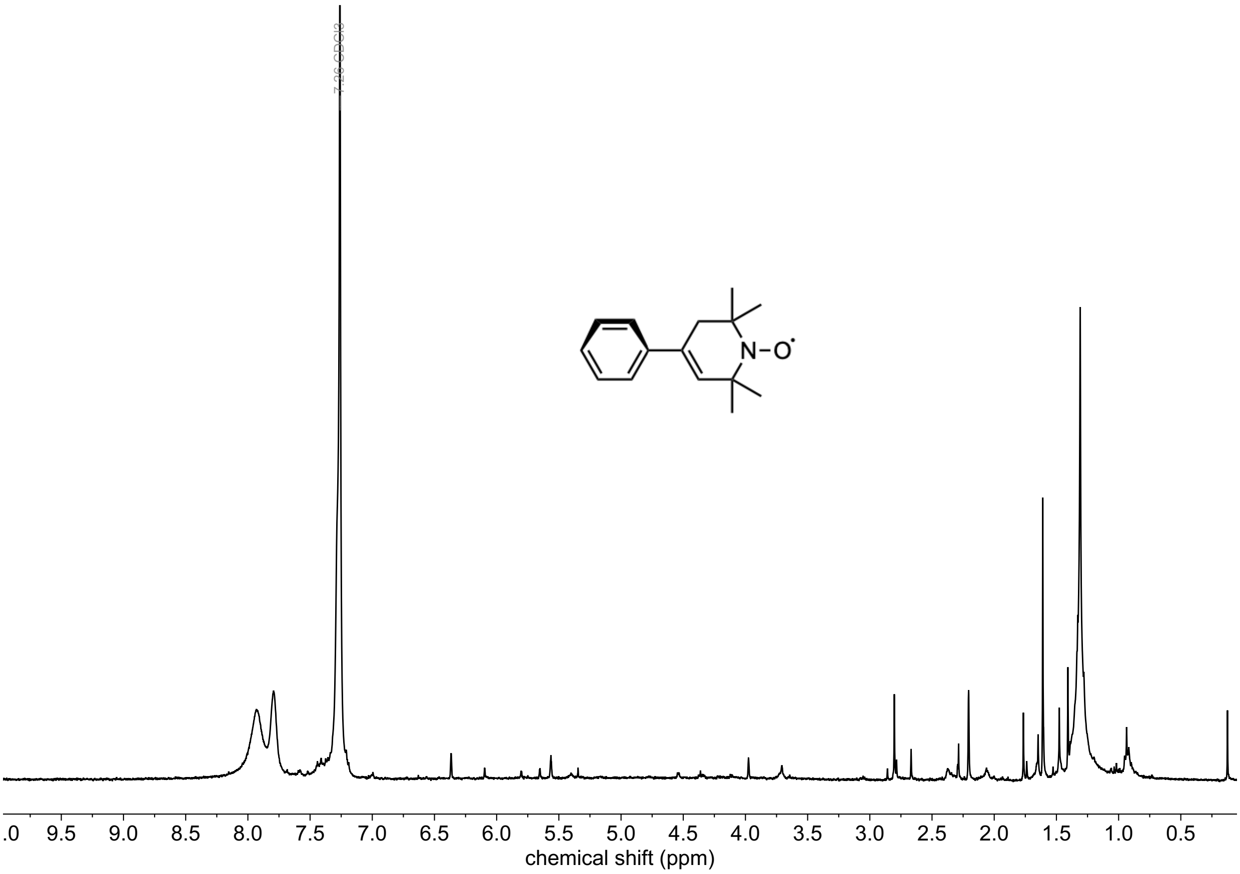

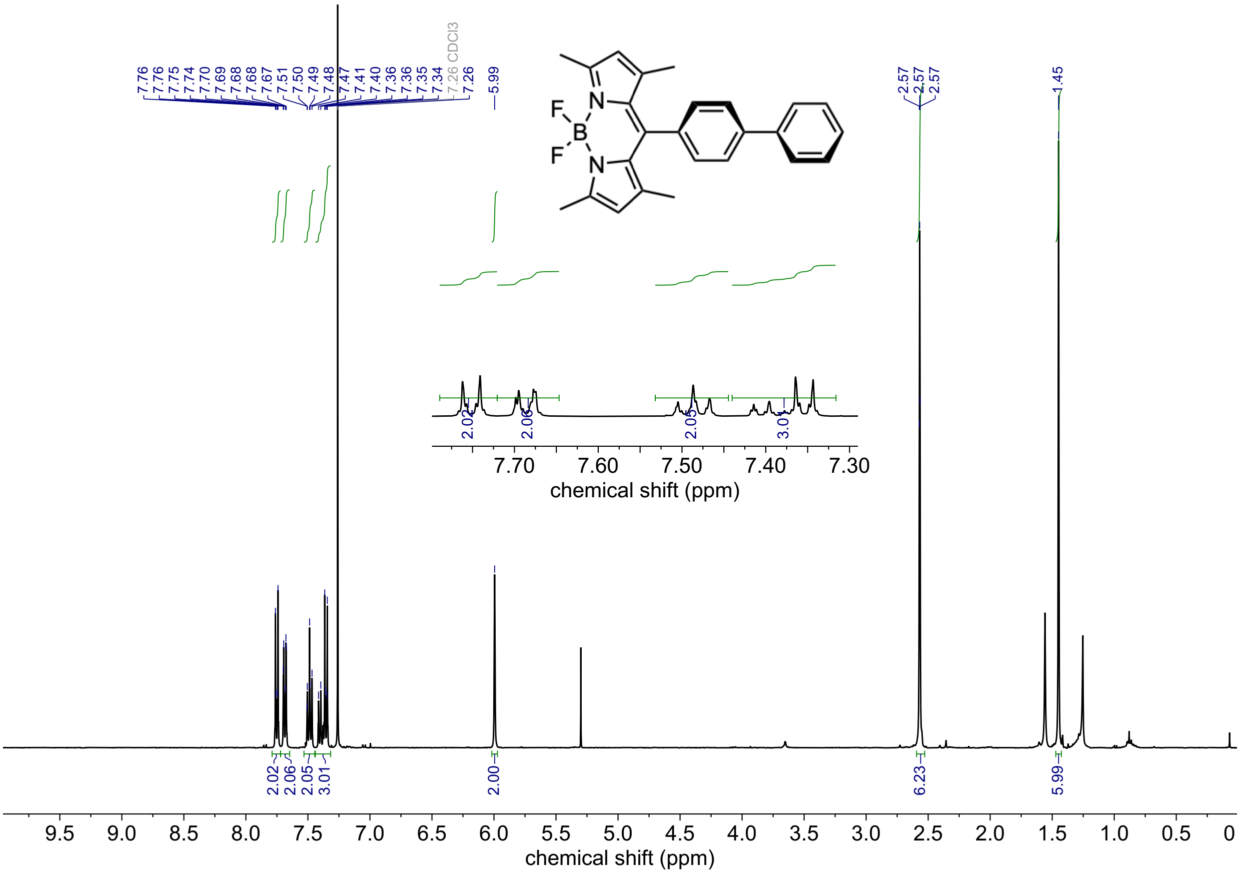

Figure S23: ^{1}H NMR spectra of compounds ph-*e*BODIPY (*top*) and BODIPY-biph (*bottom*).

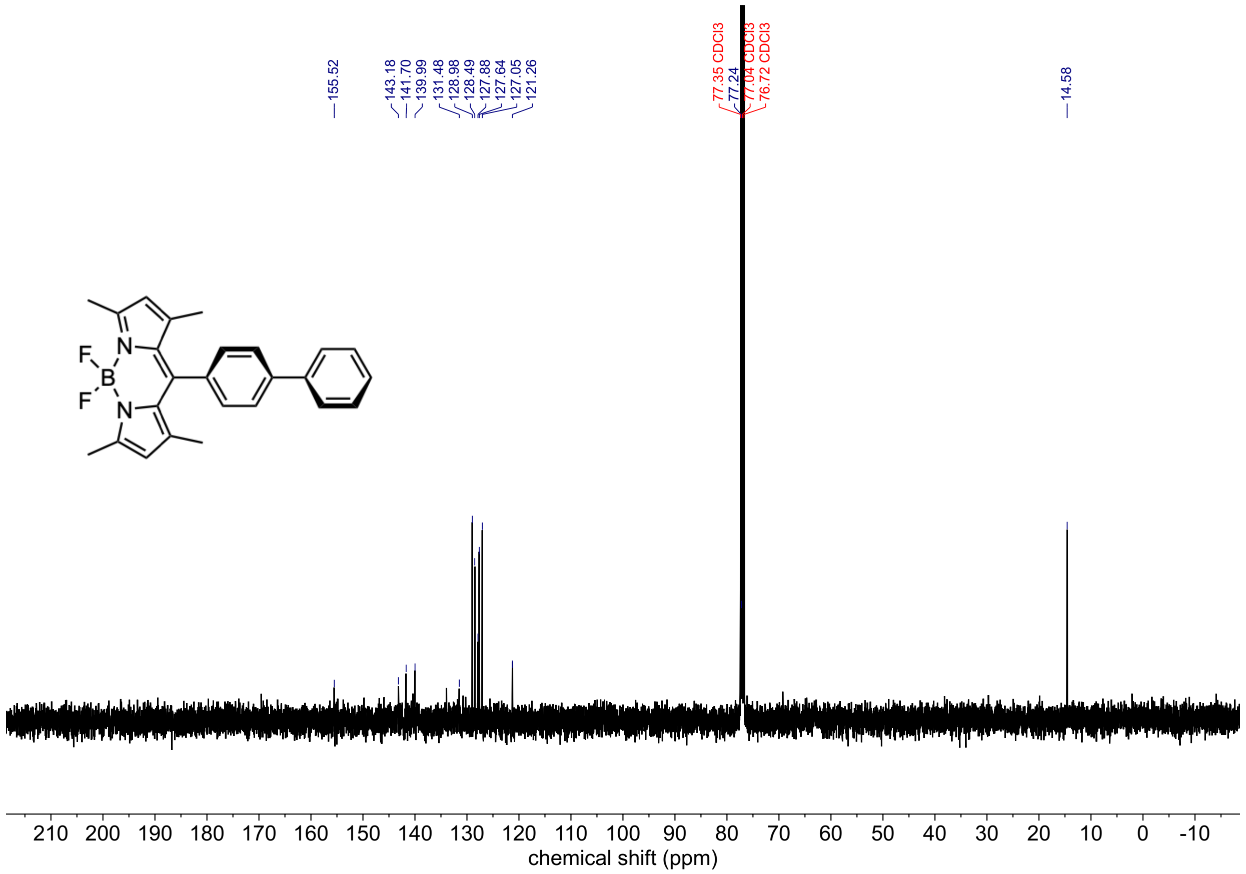

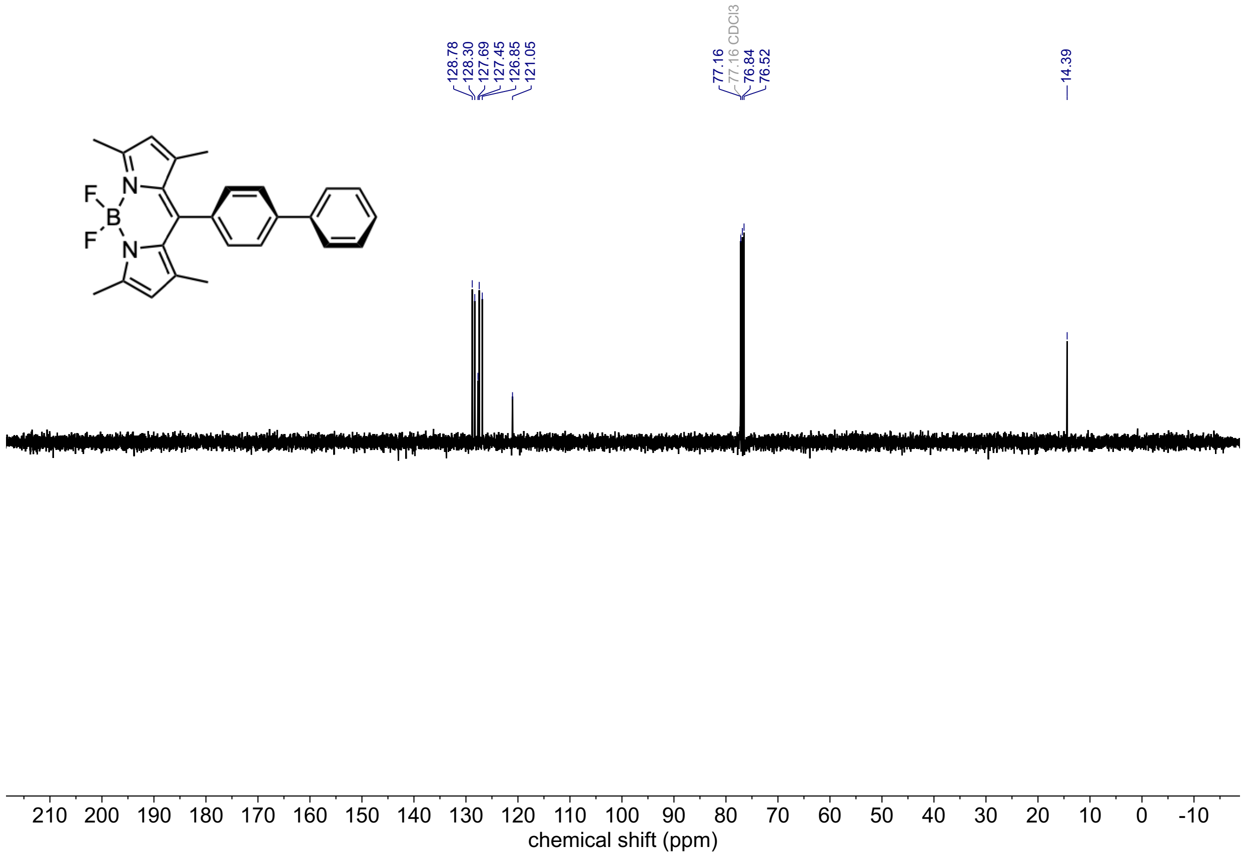

Figure S24: ^{13}C and DEPT-135 NMR spectra of compound BODIPY-biph.

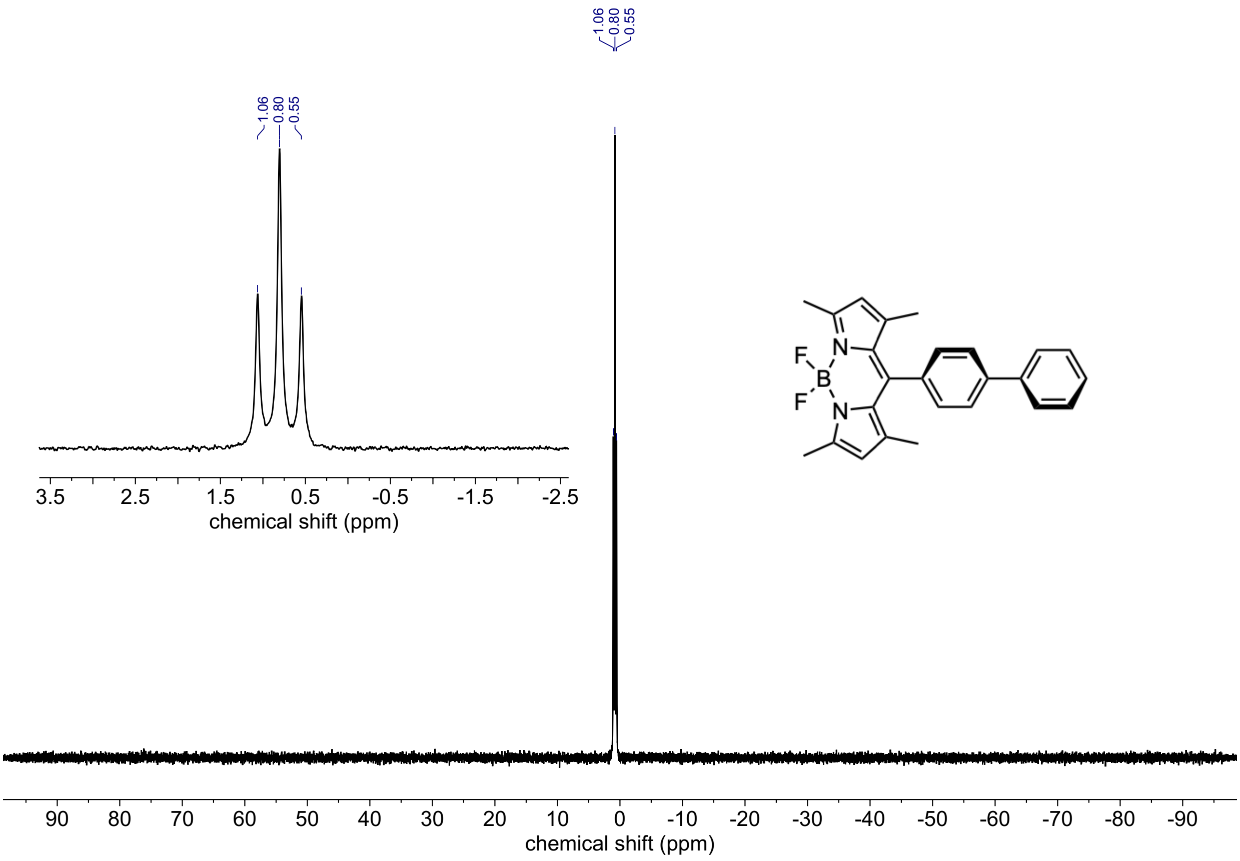

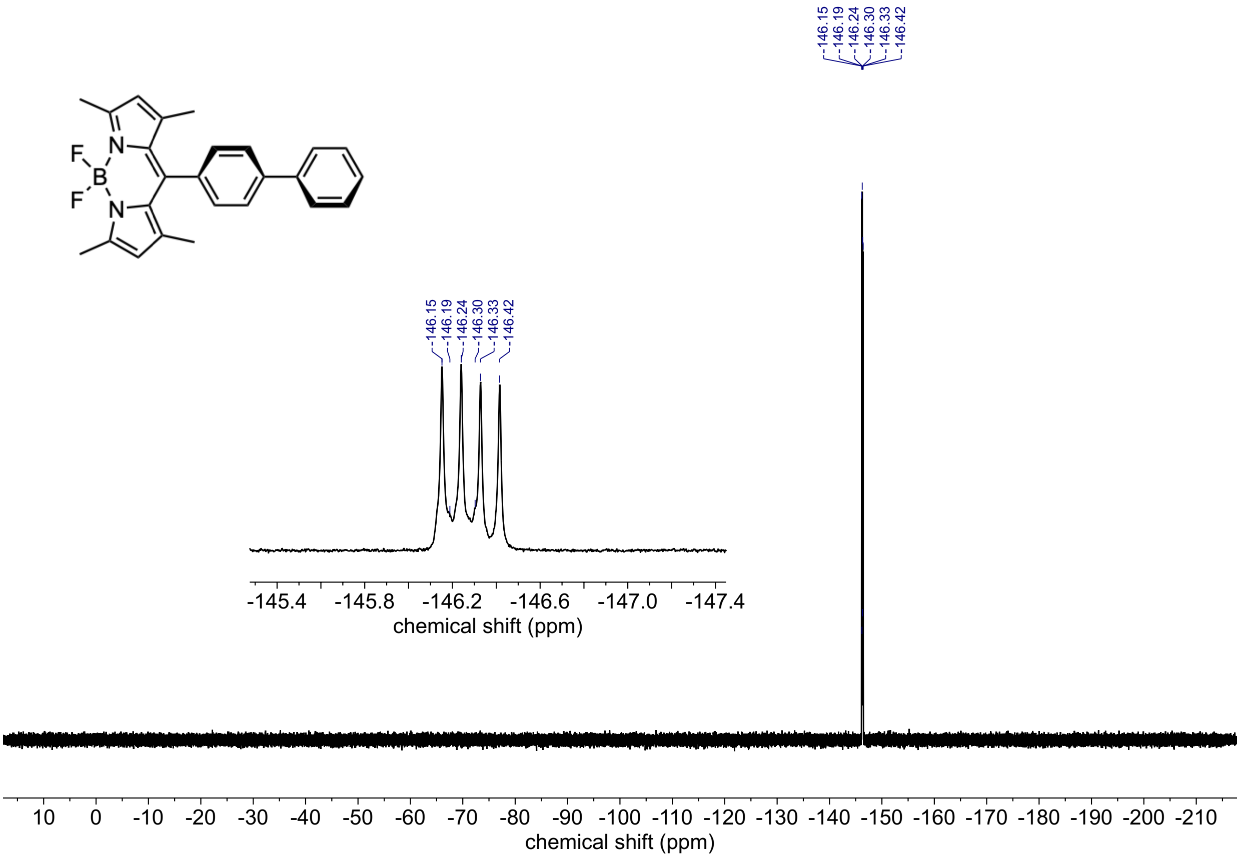

Figure S25: ^{11}B and ^{19}F NMR spectra of compound BODIPY-biph.

5 HRMS data

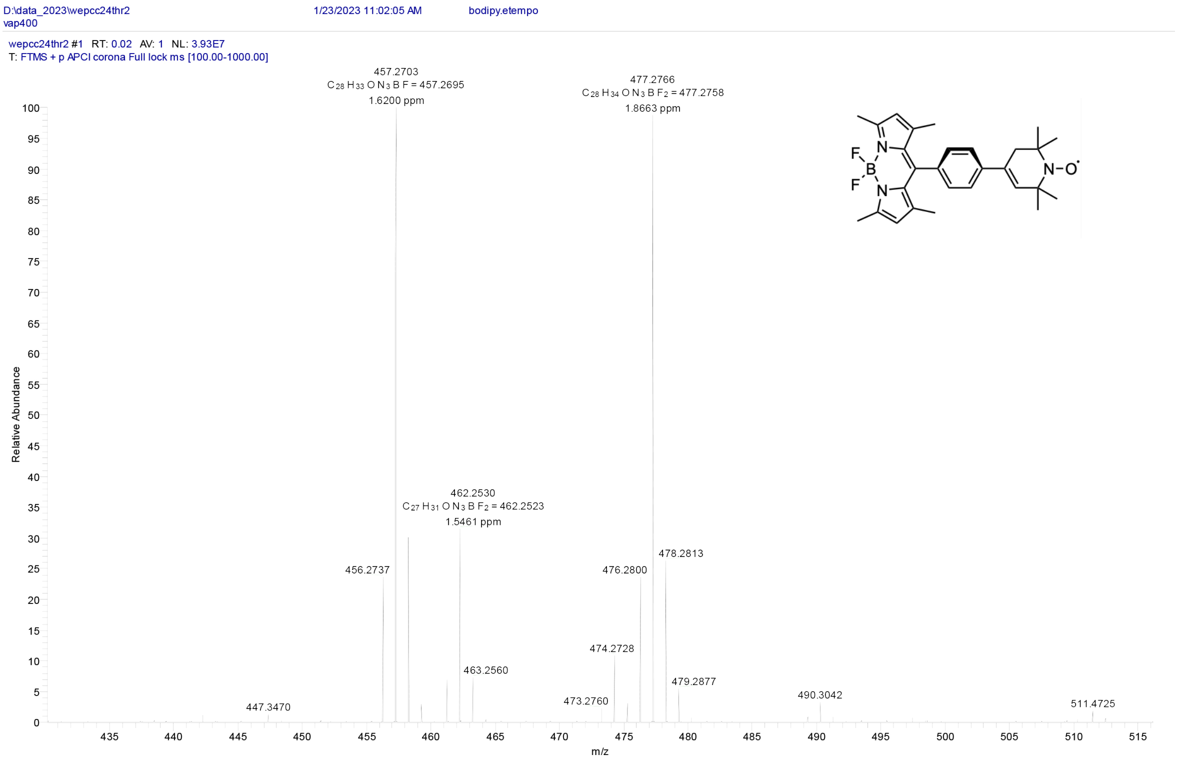

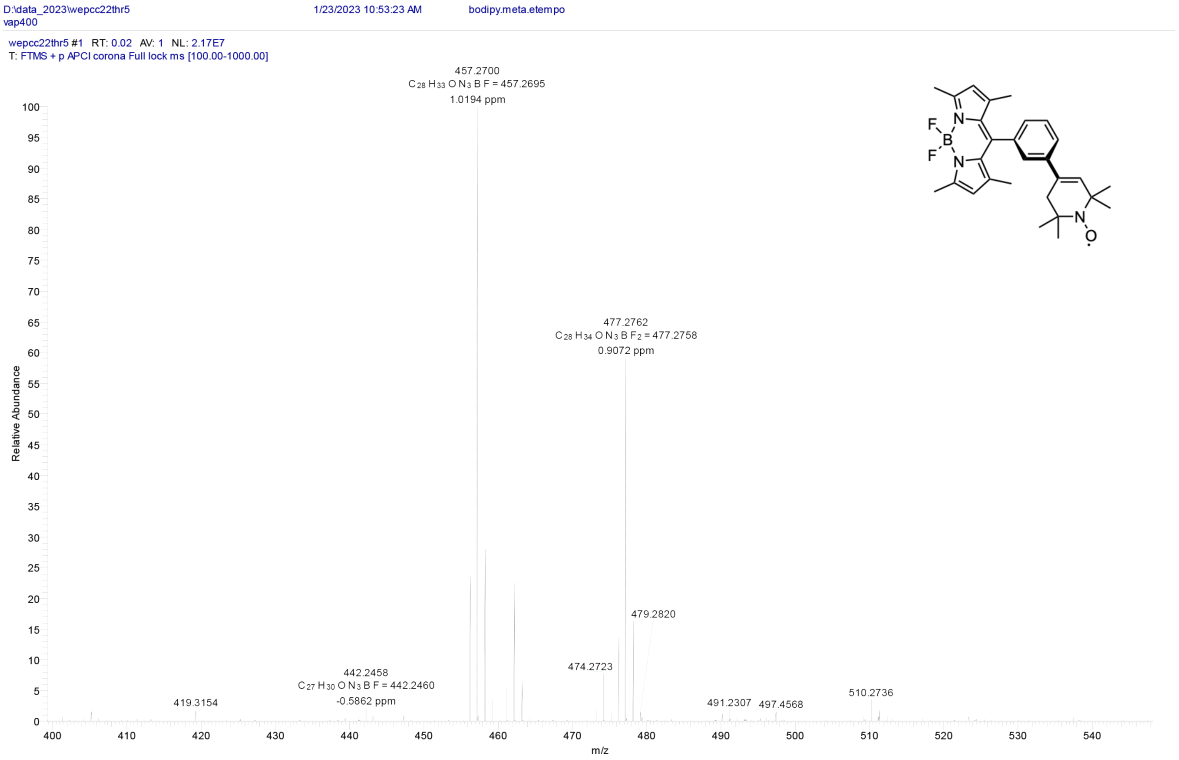

Figure S26: HRMS-ESI analysis for compounds BODIPY-*p*-*e*TEMPO (*top*) and BODIPY-*m*-*e*TEMPO (*bottom*).

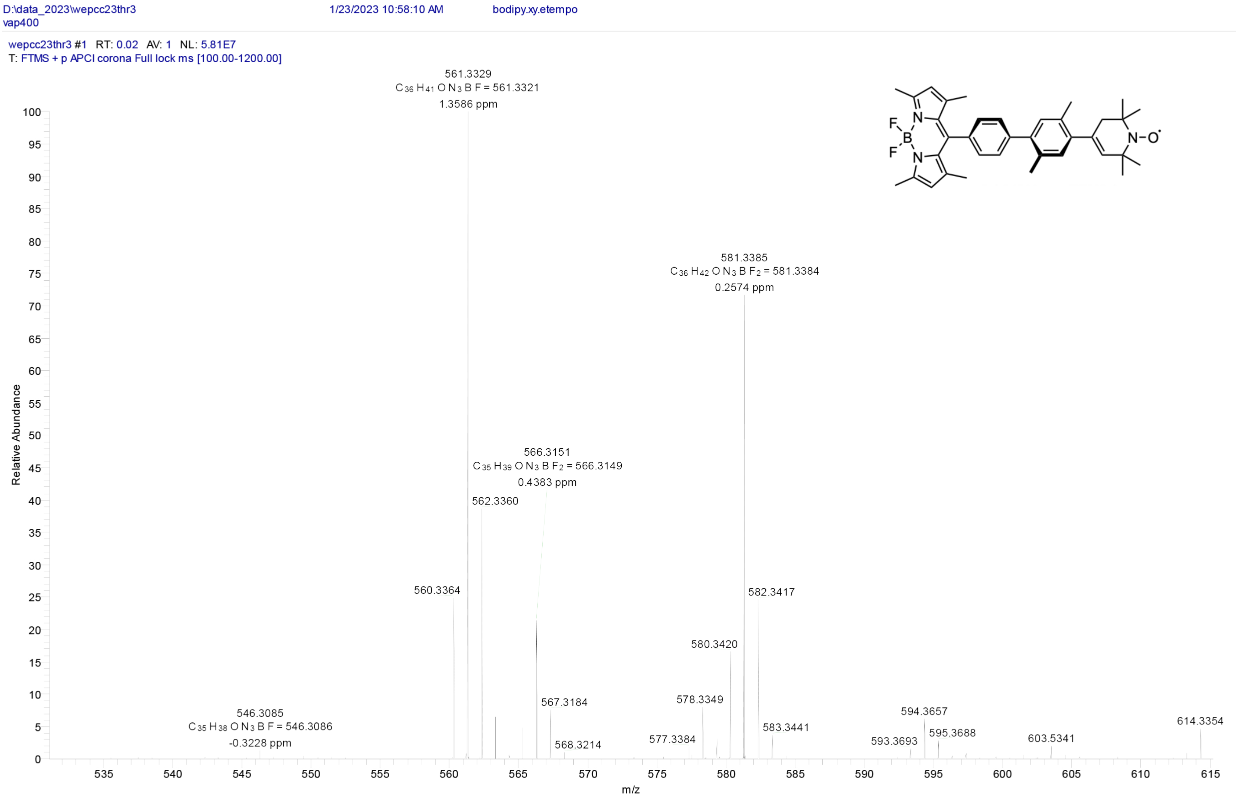

Figure S27: HRMS-ESI analysis for compound BODIPY-*xy*-*e*TEMPO.

References

[1] Exner, J.; Eusterwiemann, S.; Janka, O.; Doerenkamp, C.; Massolle, A.; Niehaus, O.; Daniliuc, C. G.; Pöttgen, R.; Neugebauer, J.; Studer, A.; Eckert, H. Antiferromagnetic ordering based on intermolecular London dispersion interactions in amphiphilic TEMPO ammonium salts. *Phys. Chem. Chem. Phys.* **2018**, *20*, 28979–28983.

[2] Kálai, T.; Jekő, J.; Berente, Z.; Hideg, K. Palladium-catalyzed cross-coupling reactions of paramagnetic vinyl bromides and paramagnetic boronic acids. *Synthesis* **2006**, *38*, 439–446.

[3] Das, G.; Cherumukkil, S.; Padmakumar, A.; Banakar, V. B.; Praveen, V. K.; Ajayaghosh, A. Tweaking a BODIPY spherical self-assembly to 2D supramolecular polymers facilitates excited-state cascade energy transfer. *Angew. Chem. Int. Ed.* **2021**, *60*, 7851–7859.

[4] Mendive-Tapia, L.; Zhao, C.; Akram, A. R.; Preciado, S.; Albericio, F.; Lee, M.; Serrels, A.; Kielland, N.; Read, N. D.; Lavilla, R.; Vendrell, M. Spacer-free BODIPY fluorogens in antimicrobial peptides for direct imaging of fungal infection in human tissue. *Nat. Commun.* **2016**, *7*, 10940.

[5] Pospiech, S.; Bolte, M.; Lerner, H.-W.; Wagner, M. Diborylated magnesium anthracene as precursor for B2H5-bridged 9,10-dihydroanthracene. *Chem. Eur. J.* **2015**, *21*, 8229–8236.

[6] Lienkamp, K.; Schnell, I.; Groehn, F.; Wegner, G. Polymerization of styrene sulfonate ethyl ester by ATRP: synthesis and characterization of macromonomers for Suzuki polycondensation. *Macromol. Chem. Phys.* **2006**, *207*, 2066–2073.

[7] Gupta, N.; Reja, S. I.; Bhalla, V.; Gupta, M.; Kaur, G.; Kumar, M. A BODIPY based fluorescent probe for evaluating and identifying cancer, normal and apoptotic C6 cells on the basis of changes in intracellular viscosity. *J. Mater. Chem. B* **2016**, *4*, 1968–1977.

[8] Wang, Z.; Zhao, J.; Barbon, A.; Toffoletti, A.; Liu, Y.; An, Y.; Xu, L.; Karatay, A.; Yaglioglu, H. G.; Yildiz, E. A.; Hayvali, M. Radical-enhanced intersystem crossing in new bodipy derivatives and application for efficient triplet-triplet annihilation upconversion. *J. Am. Chem. Soc.* **2017**, *139*, 7831–7842.

[9] Lakowicz, J. R. *Principles of fluorescence spectroscopy*; Springer: New York, 2006.

[10] Weller, A. Photoinduced electron transfer in solution: exciplex and radical ion pair formation free enthalpies and their solvent dependence. *Z. Phys. Chem.* **1982**, *133*, 93–98.

[11] Wohlfarth, C. In *Landolt-Börnstein, New Series*; Lechner, M. D., Ed.; Springer: Berlin, 2008.

[12] Marcus, R. A.; Sutin, N. Electron transfers in chemistry and biology. *Biochim. Biophys. Acta* **1985**, *811*, 265–322.

[13] Barbara, P. F.; Meyer, T. J.; Ratner, M. A. Contemporary Issues in Electron Transfer Research. *J. Phys. Chem.* **1996**, *100*, 13148–13168.

[14] Neese, F. Software update: The ORCA program system – Version 5.0. *WIREs Comput. Mol. Sci.* **2022**, e1606.

[15] Vaissier, V.; Kirkpatrick, J.; Nelson, J. Influence of polar medium on the reorganization energy of charge transfer between dyes in a dye sensitized film. *Phys. Chem. Chem. Phys.* **2013**, *15*, 4804–4814.

[16] Herb, K.; Tschaggelar, R.; Denninger, G.; Jeschke, G. Double resonance calibration of *g* factor standards: carbon fibers as a high precision standard. *J. Magn. Reson.* **2018**, *289*, 100–106.

[17] Stoll, S.; Schweiger, A. EasySpin, a comprehensive software package for spectral simulation and analysis in EPR. *J. Magn. Reson.* **2006**, *178*, 42–55.

[18] Frisch, M. J. et al. Gaussian 16 Revision C.01. 2016; Gaussian Inc. Wallingford CT.

[19] Weigend, F.; Ahlrichs, R. Balanced basis sets of split valence, triple zeta valence and quadruple zeta valence quality for H to Rn: Design and assessment of accuracy. *Phys. Chem. Chem. Phys.* **2005**, *7*, 3297.

[20] Becke, A. D. A new mixing of Hartree–Fock and local density-functional theories. *J. Chem. Phys.* **1993**, *98*, 1372–1377.

[21] Lee, C.; Yang, W.; Parr, R. G. Development of the Colle-Salvetti correlation-energy formula into a functional of the electron density. *Phys. Rev. B* **1988**, *37*, 785.

[22] Vosko, S. H.; Wilk, L.; Nusair, M. Accurate spin-dependent electron liquid correlation energies for local spin density calculations: a critical analysis. *Can. J. Phys.* **1980**, *58*, 1200–1211.

[23] Stephens, P. J.; Devlin, F. J.; Chabalowski, C. F.; Frisch, M. J. Ab initio calculation of vibrational absorption and circular dichroism spectra using density functional force fields. *J. Phys. Chem.* **1994**, *98*, 11623–11627.

[24] Neese, F.; Wennmohs, F.; Hansen, A.; Becker, U. Efficient, approximate and parallel Hartree–Fock and hybrid DFT calculations. A 'chain-of-spheres' algorithm for the Hartree–Fock exchange. *Chem. Phys.* **2009**, *356*, 98–109.

[25] Yanai, T.; Tew, D. P.; Handy, N. C. A new hybrid exchange–correlation functional using the Coulomb-attenuating method (CAM-B3LYP). *Chem. Phys. Lett.* **2004**, *393*, 51–57.

[26] Zheng, J.; Xu, X.; Truhlar, D. G. Minimally augmented Karlsruhe basis sets. *Theor. Chem. Acc.* **2011**, *128*, 295–305.

[27] Angeli, C.; Borini, S.; Cestari, M.; Cimiraglia, R. A quasidegenerate formulation of the second order *n*-electron valence state perturbation theory approach. *J. Chem. Phys.* **2004**, *121*, 4043–4049.

[28] Boys, S. F. Construction of some molecular orbitals to be approximately invariant for changes from one molecule to another. *Rev. Mod. Phys.* **1960**, *32*, 296.

[29] Franz, M.; Neese, F.; Richert, S. Calculation of exchange couplings in the electronically excited state of molecular three-spin systems. *Chem. Sci.* **2022**, *13*, 12358–12366.
